# *Lomentospora prolificans* Disseminated Infections: A Systematic Review of Reported Cases

**DOI:** 10.3390/pathogens12010067

**Published:** 2022-12-31

**Authors:** Afroditi Konsoula, Aris P. Agouridis, Lamprini Markaki, Constantinos Tsioutis, Nikolaos Spernovasilis

**Affiliations:** 1 Department of Pediatrics, General Hospital of Sitia, 72300 Sitia, Greece; 2 School of Medicine, European University Cyprus, 2404 Nicosia, Cyprus; 3 Department of Internal Medicine, German Oncology Center, 4108 Limassol, Cyprus; 4 “Iliaktida” Pediatric & Adolescents Medical Center, 4001 Limassol, Cyprus; 5 Department of Infectious Diseases, German Oncology Center, 4108 Limassol, Cyprus; 6 School of Medicine, University of Crete, 71303 Heraklion, Greece

**Keywords:** *Lomentospora prolificans*, fungal infection, dissemination, immunocompromised

## Abstract

Background: *Lomentospora prolificans*, a rare, highly virulent filamentous fungus with high rates of intrinsic resistance to antifungals, has been associated with different types of infections in immunocompromised as well as immunocompetent individuals. Objective: To systematically address all relevant evidence regarding *L. prolificans* disseminated infections in the literature. Methods: We searched Medline via PubMed and Scopus databases through July 2022. We performed a qualitative synthesis of published articles reporting disseminated infections from *L. prolificans* in humans. Results: A total of 87 studies describing 142 cases were included in our systematic review. The pathogen was most frequently reported in disseminated infections in Spain (n = 47), Australia (n = 33), the USA (n = 21), and Germany (n = 10). Among 142 reported cases, 48.5% were males. Underlying conditions identified for the majority of patients included malignancy (72.5%), hemopoietic stem cell transplantation (23.2%), solid organ transplantation (16%), and AIDS (2%). Lungs, central nervous system, skin, eyes, heart and bones/joints were the most commonly affected organs. Neutropenia was recorded in 52% of patients. The mortality rate was as high as 87.3%. Conclusions: To the best of our knowledge, this is the first systematic review conducted on disseminated infections due to this rare microorganism. Physicians should be aware that *L. prolificans* can cause a diversity of infections with high mortality and primarily affects immunocompromised and neutropenic patients.

## 1. Introduction

*Lomentospora prolificans*, formerly known as *Scedosporium prolificans* or *Scedosporium inflatum*, is a rare emerging opportunistic pathogen that primarily affects immunocompromised individuals but can also cause infections in healthy populations [1]. It is found in the environment, including soil, decaying organic matter, and contaminated water [2,3]. The first report as a pathogen in humans was in 1984, when Malloch and Salkin isolated this fungus from an immunocompetent patient with osteomyelitis [4]. 

*L. prolificans* can grow on standard mycological media such as Sabouraud’s dextrose agar (SDA) or potato dextrose agar (PDA) [5]. Characteristic macroscopic features include olive-gray to black colony morphology and susceptibility to cycloheximide [6]. Microscopic features that may indicate the presence of *L. prolificans* include visualization of flask-shaped conidiophores which are inflated or swollen at the base, from which single, or clusters of, conidia emerge [6].

*L. prolificans* infection causes a wide range of clinical manifestations from localized to disseminated infections, depending on the immune status of the infected individual [7]. Disseminated infection usually affects immunocompromised hosts and is accompanied with a high mortality rate, as highlighted in previous reviews [1,8]. 

*L. prolificans* is increasingly recognized as a cause of invasive fungal infection in geographic areas such as Australia [9], the United States [10,11], and some parts of Europe [12,13,14]. High rates of intrinsic resistance to several antifungals reduce the possibility of successful recovery [15]. The lack of or difficult access to rapid species-specific diagnostic methods further complicates the treatment of this infection [16]. 

Herein, we systematically address the literature on all relevant cases of disseminated infections caused by *L. prolificans* in humans.

## 2. Materials and Methods

### 2.1. Study Design

The purpose of this systematic review is to evaluate and better understand the clinical profile and pathogenicity of disseminated infections caused by *L. prolificans.*

We performed a qualitative synthesis of published articles reporting disseminated infection from *L. prolificans* in humans. 

### 2.2. Search Strategy

An extensive bibliographic search of Medline via PubMed and Scopus databases was conducted from inception until 31 July 2022. Only articles published in English were included. Initial searches were performed using the following search terms: *“(Lomentospora prolificans*) OR (*Scedosporium prolificans*) OR (*Scedosporium inflatum*)”. Additional studies were identified from the references provided by retrieved studies.

### 2.3. Inclusion and Exclusion Criteria

The inclusion criteria for our systematic review included articles that contained at least one case of disseminated infection with *L. prolificans*. Disseminated infection was defined as (1) clinical syndrome consistent with infection and (2) recovery of the isolate from blood samples or microbiological and/or pathological evidence of infection at ≥ 2 noncontiguous sites. Only papers based on humans and written in English were considered eligible. 

Studies were excluded if they did not fulfil inclusion criteria; if they reported only localized infection by *L. prolificans*; or if the infections were not in humans.

### 2.4. Data Extraction 

Studies were independently and thoroughly examined by two investigators (A.K., A.P.A.) and studies’ characteristics (author, year, study design, country, patient age/sex, underlying disease/conditions, clinical manifestations, sample, treatment, outcome) were extracted. Any discrepancy between the reviewers was resolved by consensus. For the review of our analysis, which was designed according to the guidelines of 2020 [17], data extraction was performed with adherence to Preferred Reporting Items for Systematic reviews and Meta-Analysis (PRISMA model). Due to the study design, no institutional Review Board approval was obtained. 

### 2.5. Assessment of Risk of Bias 

A systematic assessment of bias in the included studies was performed using the Joanna Briggs Institute (JBI) critical appraisal checklist for case reports [18]. The items used for the assessment of each study were as follows: patient’s demographic characteristics, patient’s history, patient’s current clinical condition, diagnostic tests or assessment methods and the results, the intervention(s) or treatment procedure(s), post-intervention clinical condition, adverse events (harms) or unanticipated events, takeaway lessons. According to the recommendations of the JBI tool, a judgment of “1” indicates low risk of bias, whereas a “0” on any of the included questions negatively affects the overall quality the case reports. An overall score ≤ 49% equals with high risk of bias, 50% to 69% equals with moderate risk of bias, and ≥ 70% equals with low risk of bias. Risk-of-bias assessment was performed independently by 2 reviewers (A.K., A.P.A.); disagreements were resolved by consensus.

### 2.6. Statistical Analysis

Associations of survival with surgery and neutropenic/immunosuppressant patients were assessed using the Chi-square test (χ^2^). Statistical significance was set at 5% significance level (*p* < 0.05). Data were processed and analyzed using IBM SPSS Statistics for Windows, Version 29.0 (Armonk, NY, USA: IBM Corp, USA).

## 3. Results

### 3.1. Study Selection

**Figure 1:**
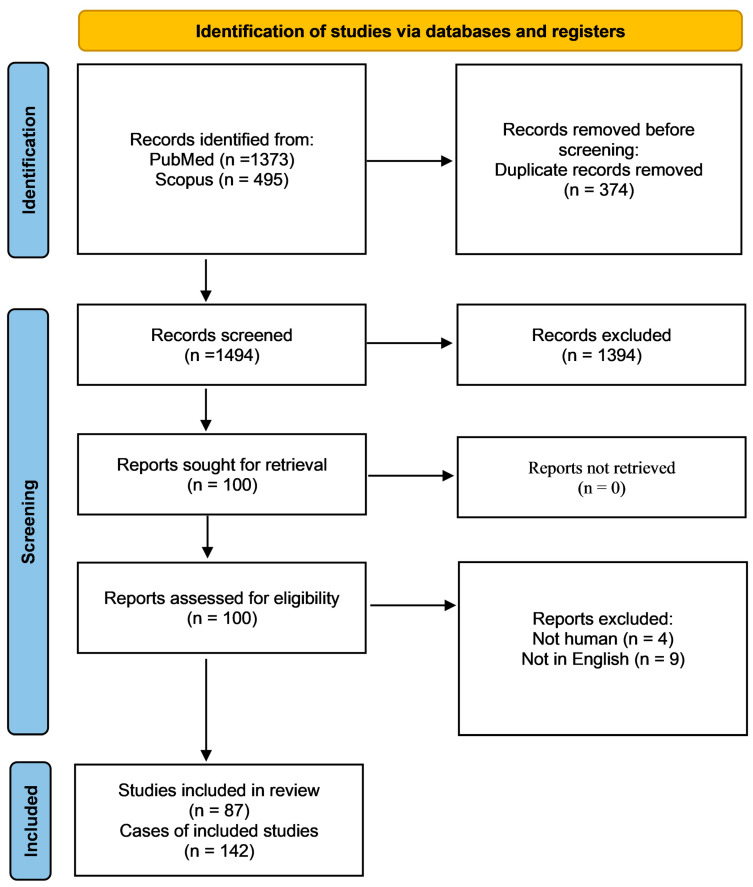
PRISMA flow diagram of article selection process.

In Figure 1, the PRISMA flow chart reveals the selection process of included studies. With the above-mentioned search terms, we identified 1373 records on Medline via PubMed and 495 additional records on Scopus. After detecting and removing duplicates, 1494 articles remained, among which we initially excluded 1394 because of study design. Subsequently, we examined in detail the remaining 100 articles. Among them, 13 studies were rejected because selection criteria were not met (Supplementary Table S1 and Figure 1). Finally, 87 studies with a total of 142 cases (patients with disseminated *L. prolificans* infection) were included in our systematic review.

### 3.2. Study Characteristics

The included studies were published between 1990 and 2022 (Table 1). A total of 142 individual cases from 87 publications of disseminated infection by *L. prolificans* fulfilled the inclusion criteria. Studies were more frequently reported in Spain (n = 41), Australia (n = 33), the USA (n = 21), Germany (n = 10), Japan (n = 8), USA/Spain (n = 6), France (n = 6), Mexico (n = 6), The Netherlands (n = 2), Canada (n = 2), South Korea (n = 1), Italy (n = 1), Brazil (n = 1), Belgium (n = 1), Thailand (n = 1), Poland (n = 1), and India (n = 1). Among a total of 127 adults, 5 children (defined as patients <16 years old), and 10 patients whose age was not specified, males represented 48.5% and females 45%, while in 6.3% sex was not mentioned. Underlying conditions, identified for the majority of patients, included malignancy (72.5%), hemopoietic stem cell transplantation (HSCT) (23.2%), solid organ transplantation (16%), and AIDS (2%). No underlying condition was reported in four patients. Neutropenia was recorded in 52% of patients. Lungs, central nervous system (CNS), skin, eyes, heart and bones/joints were the most commonly affected organs. Blood cultures were positive in 107 of 142 (75.3%) patients. The majority of patients systematically received amphotericin B, voriconazole, terbinafine, itraconazole, and fluconazole either as monotherapy or in combination therapy. The overall mortality rate was 87.3% (Table 1). 

### 3.3. Clinical Outcomes

After performing the Chi-Square test, an association between surgery and survival was observed (Pearson Chi-Square = 21.044, *p* < 0.001). More specifically, patients who underwent surgery had a 11.329 times higher probability of surviving [95% CI, (3.388–37.881)]. Moreover, we found that immunocompetent patients had a 10.3 [95% CI, (1.333–83.333)] higher probability of surviving compared with neutropenic/immunosuppressant patients (Pearson Chi-Square = 7.320, *p* = 0.05).

### 3.4. Quality Appraisal

The overall quality was very good, as 72 articles had a low risk of bias, while 9 studies had a high risk of bias and 6 studies had a moderate risk of bias. Quality appraisal results are presented in Supplementary Table S2.

## 4. Discussion

The current systematic review focuses on disseminated infections caused by *L. prolificans* in humans. To the best of our knowledge, this is the first systematic review conducted on disseminated infections due to this rare microorganism. 

**Figure 2:**
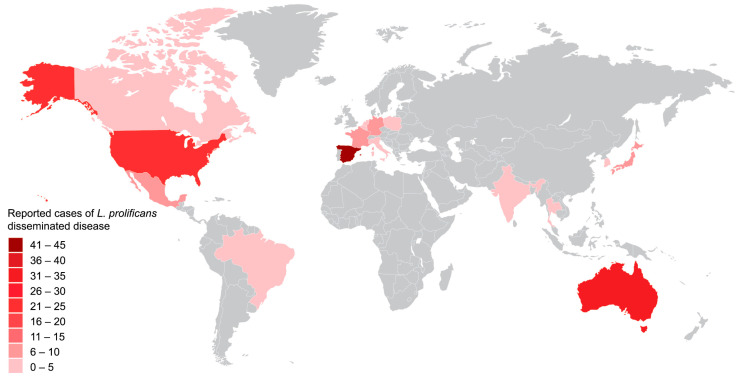
Worldwide distribution of *L. prolificans* disseminated disease.

*L. prolificans* is a rare filamentous fungus found primarily in the environment, including soil, decaying organic matter, and contaminated water [2,3]. Regarding the epidemiology of *L. prolificans* disseminated infection, cases were initially reported in the dry climates of Spain, Australia and the southwestern United States. Recently, however, there have been publications from other countries, specifically Germany, Japan, France, Mexico, The Netherlands, Canada, South Korea, Italy, Brazil, Belgium, Thailand, Poland, and India (Figure 2). Excluded studies due to different language concern cases reported in the aforementioned countries (Supplementary material).

This pathogen can infect both immunocompetent and immunocompromised patients and thus acts both as a primary and an opportunistic pathogen [100]. Skin, soft tissue, muscle, bone, and joint infections are more common in immunocompetent hosts, and infection usually requires disruption of the anatomic barrier by trauma, surgery, or corticosteroid injections [1,101]. Almost all cases presented in this review involve diseases and conditions indicative of severe immunosuppression. Airway colonization is common in patients with cystic fibrosis and lung transplantation [1,102,103]. Structural changes in the airways, long-term immunosuppression, and previous exposure to antifungal drugs contribute to the higher prevalence of *L. prolificans* in these patient populations [102,103,104]. 

Disseminated infection is the most common pattern of *L. prolificans* infection reported, and is associated with very high mortality rate, as shown in our systematic review. Risk factors for dissemination include solid organ transplantation, HSCT, malignancies (especially hematologic), AIDS, neutropenia, and immunosuppressive therapy [1,8,105,106]. The primary location of the fungus, the degree of immunosuppression, and the speed of disease progression determine the clinical outcome. Primary location of the fungus, such as eyes, joint, bone, and skin plays an important role in clinical outcome, since resection of surgically amenable lesions is significantly associated with improved survival [105,107]. This comes in agreement with our results, since those patients who underwent surgery had higher survival rate. The most frequent clinical manifestations of disseminated disease include fever and CNS, heart and/or respiratory involvement, along with skin lesions, particularly numerous erythematous non-pruritic skin nodules with or without a necrotic center [1,7,44]. 

Several determinants of pathogenesis have a role in the manifestation of disease [100], associated with germination [108], biofilm formation [109], destruction of lung epithelial cells [109], and infiltration of blood vessels [110], resulting in widespread dissemination to distal organs [110]. Important molecules in the fungal cell wall that enhance fungal virulence include peptidorhamnomannan, glucosylceramide, and melanin [111]. The susceptibility of this fungus to innate immunity, particularly to neutrophils, may explain the high rate of prevalence in neutropenic patients [106]. Therefore, correction of neutropenia is of paramount importance, associated with a favorable outcome [26]. At the same time, a weak innate systemic response of microglial cells in the CNS explains the propensity of this fungus to invade and live in the CNS, a phenomenon known as neurotropism [112]. Detection of *L. prolificans* in clinical specimens relies principally on direct microscopic examination of fresh specimens or histopathologic analysis, together with culture on appropriate culture media [5]. Histopathologic examination can provide valuable evidence of invasive disease, but culture is necessary because different molds share the same characteristics under the microscope [5]. Direct microscopy and culture are the cornerstone of proven fungal infection [113]. A positive culture from the respiratory system in the absence of radiologic or endobronchial changes may indicate colonization [114]. Disseminated infection can be detected with blood cultures. Positive blood culture is rare in most molds, except those capable of angioinvasion with widespread dissemination, such as *Scedosporium*/*Lomentospora* and *Fusarium* species, and zygomycetes such as *Rhizopus* and *Mucor* [110] As shown in this systematic review, blood cultures were positive in 107 of 142 (75.3%) patients. However, their diagnostic utility is limited because most blood cultures become positive late in the course of the disease due to slow growth of the microorganism [1]. Molecular techniques, such as PCR, either panfungal or species-specific, followed by DNA sequencing, can detect invasive fungal infections directly from fresh and formalin-fixed paraffin-embedded (FFPE) material, but only in conjunction with histopathologic examination [115,116,117]. Several case reports have mentioned high serum 1, 3-beta-D-glucan (BDG) levels in patients with *L. prolificans* infection [51,80], while some other reports, mentioned low serum BDG levels [118]. Hence, although this panfungal biomarker (BDG) may be useful in diagnosis when invasive fungal infection is suspected [5], its clinical utility is controversial. Therefore, results should always be interpreted in conjunction with the other diagnostic methods mentioned above. Matrix-assisted laser desorption/ionization time-of-flight is rapid and reliable method for identifying *L. prolificans*, but is used by only few laboratories [119,120].

Treatment of *L. prolificans* infection is challenging because this fungus has intrinsic resistance to most antifungal agents used in clinical practice. The treatment strategy for disseminated disease includes a combination of surgical and antifungal therapy, as well as correction of underlying immune deficiencies [121]. Once invasive *L. prolificans* is suspected or confirmed, surgical removal of infected tissue should be initiated if feasible [121]. Current clinical practice guidelines recommend that first-line antifungal treatment with voriconazole and terbinafine plus or minus other antifungal agents over a period of at least 4 to 6 months is associated with a favorable outcome [121]. According to Jenks et al., combination therapy with voriconazole plus terbinafine may be associated with improved treatment outcomes compared with other antifungal regimens for the treatment of invasive *L. prolificans* infections [122]. Clinical evaluation, laboratory studies (inflammatory markers, microbiologic studies), and imaging should be reviewed frequently to assess respond to treatment. Frequency depends on the concomitant conditions, disease severity and initial response to treatment. 

Inherent resistance to most available treatments raises the need for new classes of antifungal agents [123]. Olorofim, a key enzyme in the biosynthesis of pyrimidines, has the ability to inhibit dihydroorotate dehydrogenase [124]. It is currently in Phase IIB clinical trials for the treatment of invasive mold infections, including *L. prolificans*, in patients with limited treatment options [124]. The efficacy of olorofim has been demonstrated in in vitro studies and improved clinical outcomes have been observed in two case reports [124,125,126].

This study has several limitations. It was not possible to perform a meta-analysis because all data are based on case reports and small case series. The above limitations could have affected the quality of our findings and conclusions. However, by using the JBI critical appraisal checklist for each article included in our systematic review, we attempted to minimize the risk of bias and increase quality. The geographic distribution of publications that were included in our review probably reflects research and clinical interest rather than presence of the fungus only in these areas and environments. Finally, despite the high number of titles analyzed in our review, several studies on invasive infections by *L. prolificans* were excluded, as they did not fulfil inclusion criteria. Although excluded, these studies provide important clinical information on these infections [8,102,107,122].

## 5. Conclusions

Disseminated disease caused by *L. prolificans* is a rare infection with significant mortality, and should be suspected especially in immunocompromised and neutropenic patients. Early diagnosis and careful interpretation of culture results are important in the management of these patients. Novel antifungal agents and further exploration of therapeutic options are needed to improve the outcome of this highly fatal infection. Healthcare providers treating patients with disseminated fungal infection should be aware of this life-threatening pathogen. 

## Figures and Tables

**Table 1:** Study characteristics of *Lomentospora prolificans* infections reported in the literature.

Author	Year	Study Design	Country	Patient Age/Sex	Underlying Disease/Conditions	Clinical Manifestations	Sample	Treatment	Outcome
Aldoss [19]	2019	Retrospective cohort study	USA	55/F	AML, alloHSCT	Fungemia	Blood culture	POS	NA
Alvarez [14]	1995	Case series	Spain	27/F	AML	Pneumonia, fungemia	Blood culture	AMB	Died
Alvarez [14]	1995	Case series	Spain	45/F	AML, neutropenia	Pneumonia, fungemia	Blood culture	AMB	Died
Alvarez [14]	1995	Case series	Spain	79/F	ALL, neutropenia	Pneumonia, pleural effusion, fungemia	Blood culture	AMB	Died
Alvarez [14]	1995	Case series	Spain	54/F	AML, neutropenia	Pneumonia, fungemia	Blood, tracheal aspirate culture	AMB	Died
Álvarez-Uría [20]	2020	Case series	Spain	25/M	Heart transplantation	Fungemia, CNS, skin, lung involvement	Blood, skin, sputum culture	VRC + TRB	Died
Ananda-Rajah [21]	2008	Case report	Australia	58/M	ALL, neutropenia	Pneumonia, fungemia, embolic skin lesions	Blood culture	VRC + TRB	Died
Balandin [22]	2016	Case report	Spain	27/M	CF, lung transplantation	Pneumonia, pleural empyema, pulmonary embolism, mycotic emboli	BAL, pleural fluid culture, thrombus sample with fungal elements	VRC + TRB + CAS + intrapleural/neb VRC POS + MTF +ANF	Died
Barbaric [23]	2001	Case series	Australia	10/F	ALL, neutropenia	Pneumonia, fungemia, skin lesions	Skin biopsy, catheter tip, blood culture	AMB + G-CSF	Died
Berenguer [12]	1997	Case series	Spain	56/M	Acute leukaemia, neutropenia	Pneumonia, fungemia	Blood, respiratory cultures	AMB + ITC	Died
Berenguer [12]	1997	Case series	Spain	52/M	Acute leukaemia, neutropenia	Fungemia, lung, eye involvement	Blood culture	FLC	Died
Berenguer [12]	1997	Case series	Spain	48/M	Acute leukaemia, neutropenia	Pneumonia, skin lesions	Skin, bone culture	AMB + FLC + Surgery	Survived
Boan [24]	2020	Case series	Australia	71/F	CLL	Pneumonia	Urine, sputum culture	VRC + TRB + ANF + L-AMB	Died
Boan [24]	2020	Case series	Australia	63/M	AML, neutropenia	Pneumonia	Blood culture	VRC + TRB + ANF	Died
Boan [24]	2020	Case series	Australia	25/F	AML, alloHSCT, neutropenia	Fungemia, osteomyelitis	Blood, sternoclavicular joint tissue, urine culture	VRC + TRB + ANF + MTF + Surgical debridement of sternoclavicular joint	Died
Boglione-Kerriena [25]	2019	Case report	France	61/NA	MM, autoHSCT	Fungemia, eye infection, meningitis, brain abscess, calculus pyelonephritis	Blood, urinary tract stone culture	VRC + TRB + MTF + intravitreal VRC + Surgical removal of the urinary stone	Died (not related to the fungal infection)
Bouza [26]	1996	Case report	Spain	74/F	AML, neutropenia	Fungemia, pneumonia, skin lesions	Blood, skin biopsy culture	AMB + G-CSF + ITC	Survived
Buil [27]	2020	Case report	The Netherlands	NA/F	-	Fungemia	Blood, stool culture	NA	Died
Chiam [28]	2013	Case report	Australia	9/F	AML, neutropenia, BMT	Endophthalmitis, fungemia	Blood culture	AMB + G-CSF + intravitreal VRC VRC + CAS + TRB + MTF + Vitrectomy	Survived
Cobo [29]	2017	Retrospective cohort study	Spain	53/M	AML, neutropenia	Fungemia	Blood culture	VRC + TRB	Died
Cooley [9]	2007	Case series	Australia	NA	ALL, alloHSCT, neutropenia	Fungemia, septic arthritis	Blood, synovium cartilage, prostate culture	NA	Died
Cooley [9]	2007	Case series	Australia	NA	AML, alloHSCT	Pneumonia, fungemia	Blood, BAL, lung, sputum culture	NA	Died
Cooley [9]	2007	Case series	Australia	NA	NHL, alloHSCT, neutropenia	Fungemia	Blood culture	None	Died
Cooley [9]	2007	Case series	Australia	NA	AML, alloHSCT	Fungemia	Blood, BAL, lung, skin culture	ITC + AMB	Died
Cooley [9]	2007	Case series	Australia	NA	MDS	Sinusitis	Sputum, maxillary sinus, pericardium, myocardium, kidney, skin, lung culture	ITC + TRB + Surgery	Died
Cooley [9]	2007	Case series	Australia	NA	AML, neutropenia	Chest wall cellulitis, skin nodules, soft tissue infection	Chest wall, Hickman catheter culture	VRC + TRB + Surgery	Survived
Damronglerd [30]	2014	Case report	Thailand	17/M	MDS, AML, neutropenia	Skin lesions, pneumonia, sinusitis, fungemia	Skin biopsy, sinus, tracheal suction, blood culture	VRC + TRB	Died
de Battle [31]	2000	Case report	Spain	45/Μ	Acute multilinear leukaemia, neutropenia	Fungemia, mycotic emboli, pneumonia, pleuritic effusion, skin lesion	Blood culture	AMB	Died
DeSimone [10]	2021	Retrospective cohort study	USA	59/M	Lung transplantation	Skin and subcutaneous infection, fungemia	Blood, urine, bilateral lower extremity skin (autopsy), lung (autopsy) culture	VRC + MICA + Surgical debridement	Died
DeSimone [10]	2021	Retrospective cohort study	USA	56/F	Lung transplantation	Endophthalmitis, septic arthritis	Bilateral knee synovial tissue, right ankle joint aspiration, aorta (autopsy) culture	CAS + VRC + AMB + TRB + ALB + Surgical debridement	Died
Elsayed [32]	1999	Case report	Canada	28/F	ALL, neutropenia	Fungemia	Blood culture	FLC + AMB	Died
Farag [33]	1992	Case report	Australia	72/F	NHL, neutropenia	Fungemia, skin lesions	Blood, CSF culture	AMB + FCS	Died
Feltkamp [34]	1997	Case report	The Netherlands	42/M	AML, neutropenia	Fungemia, pneumonia, brain emboli, skin lesions	Blood, CSF, BAL, sputum, skin biopsy culture	AMB + FCS	Died
Gosbell [35]	1999	Case series	Australia	68/M	AΜL, neutropenia	Fungemia, pneumonia	Nasal swab, blood culture	AMB	Died
Gosbell [35]	1999	Case series	Australia	33/F	AML, neutropenia	Fungemia, pneumonia, meningoencephalitis, endophthalmitis, renal/myocardial/brain abscesses, mycotic aneurysm	Blood, CSF culture	L-AMB + ITC + FLC + AMB (intraocular injection)	Died
Gosbell [35]	1999	Case series	Australia	48/F	AΜΜL, neutropenia	Fungemia, skin lesions	Blood, skin lesion culture	L-AMB (only one dose given)	Died
Gosbell [35]	1999	Case series	Australia	46/M	NHL, neutropenia	Fungemia, pneumonia	Blood culture	AMB	Died
Gow-Lee [36]	2021	Case report	USA	63/M	NHL, neutropenia, autoHSCT	Pneumonia, fungemia, septic arthritis	BAL, blood, synovial fluid culture	VRC + MICA + TRB + GM-CSF + L-AMB + Surgical debridement/amputation	Died
Grenouillet [37]	2009	Case series	France	68/M	NHL, neutropenia	Fungemia, pneumonia	Sputum, blood culture	AMB + ITC	Died
Grenouillet [37]	2009	Case series	France	44/M	CML, alloHSCT	Fungemia, gingival abscess	Gingival abscess, blood, urine, trachea culture	VRC + TRB	Died
Grenouillet [37]	2009	Case series	France	67/M	NHL, alloHSCT	Fungemia, pneumonia	Blood, urine, BAL culture	VRC + CAS	Died
Guerrero [38]	2001	Case series	Spain	45/F	AML, neutropenia	Fungemia, skin lesions, pneumonia	Blood culture	None	Died
Guerrero [38]	2001	Case series	Spain	64/M	AML, neutropenia	Fungemia, pneumonia, cerebral abscesses	Blood culture	AMB + ITC	Died
Guerrero [38]	2001	Case series	Spain	27/F	AML, neutropenia	Fungemia, pneumonia, pleural effusion, meningoencephalitis, skin lesions	Blood culture	L-AMB + ITC	Died
Guerrero [38]	2001	Case series	Spain	72/F	AML, neutropenia	Fungemia, pneumonia	Blood culture	L-AMB + ITC	Died
Guerrero [38]	2001	Case series	Spain	72/F	AML, neutropenia	Fungemia	Blood culture	None	Died
Hanmantgad [39]	2017	Case report	USA	71/M	AML, neutropenia	Fungemia	Blood culture	G-CSF	Died
Howden [40]	2003	Case report	Australia	53/F	MM, BMT, neutropenia	Sinusitis, osteomyelitis, discitis, aneurysm	Sinus, lumbar spine, hepatic artery wall culture	ITC + Surgical decompression of sinusitis ITC +TRB + Laminectomy/surgical debridement VRC + TRB + GM-CSF + Excision of hepatic artery aneurysm	Survived
Jain [41]	2017	Case report	USA	65/M	AML, neutropenia	Pneumonia, fungemia, skin lesions	Respiratory, blood, scrotal lesion culture	L-AMB + POS + ISA	Died
Kimura [42]	2010	Case report	Japan	58/F	AML, neutropenia	Pneumonia, fungemia	Blood culture	MICA + G-CSF	Died
Kubisiak-Rzepczyk [43]	2013	Case report	Poland	21/F	ALL, alloHSCT	Fungemia	Blood culture	VRC	Died
Maertens [44]	2000	Case report	Belgium	77/M	AML, neutropenia	Pneumonia, renal abscess, skin lesions	BAL, abscess culture	AMBITC + Vitrectomy	Died
Marin [45]	1991	Case report	Spain	66/M	AML, neutropenia	Pneumonia, fungemia, endophthalmitis, skin lesions	Skin lesions, blood, urine, vitreous culture	AMB	Died
Westerman [46]	1999	Case report	Australia	65/F	AML, neutropenia	Fungemia	Blood, sputum, faecal culture	AMB	Died
McKelvie [47]	2001	Case report	Australia	59/M	AML, neutropenia	Endophthalmitis, fungemia, pneumonia	Blood culture	Intravitreal AMB + AMB + VRC	Died
Nambiar [48]	2017	Case report	USA	65/M	NHL, neutropenia	Fungemia	Blood culture	None	Died
Nenoff [49]	1996	Case report	Germany	60/M	AIDS, Burkitt lymphoma, neutropenia	-	Kidney, spleen, myocardium tissue autopsy culture	FLC + G-CSF	Died
Nielsen [50]	1993	Case report	USA	17/M	AML, neutropenia	Fungemia, pneumonia, skin lesions	Blood, skin, lung tissue culture	AMB	Died
Nishimori [51]	2014	Case report	Japan	71/F	AML, neutropenia	Fungemia	Blood, fecal culture	MICAL-AMB	Died
Penteado [52]	2018	Case report	Brazil	17/M	X-linked chronic granulomatous disease, AlloHSCT	Fungemia, pneumonia	Blood, urine culture	VRC	Died
Pickles [53]	1996	Case series	Australia	41/M	AML, neutropenia	Pneumonia	Kidney, lung, liver autopsy culture	AMB	Died
Rabodonirina [54]	1994	Case report	France	50/F	Lung transplantation	Fungemia, pleural effusion, pneumonia	Pleural fluid, central venous catheter, blood culture	AMB	Died
Reinoso [55]	2013	Case report	Spain	35/F	AML, neutropenia	Fungemia, pneumonia, pleural effusion, endophthalmitis, orbital cellulitis	Vitreous fluid culture/PCR, blood culture	VRC + TRB + Vitrectomy	Died
Rivier [56]	2011	Case report	France	70/M	MDS, AML, neutropenia	Fungemia	Sputum, blood culture	G-CSFPOSCAS	Died
Rolfe [11]	2014	Case series	USA	44/M	AML, alloHSCT	Fungemia	BAL, blood, skin culture	VRC + AMB	Died
Salesa [57]	1993	Case report	Spain	56/F	AML, autoHSCT, neutropenia	Fungemia, skin lesions	Blood culture	AMB + GM-CSF	Died
Simarro [58]	2001	Case report	Spain	34/F	AML, neutropenia	Fungemia, pneumonia	Blood culture	L-AMB	Died
Simarro [58]	2001	Case report	Spain	20/F	ALL, neutropenia	Fungemia	Blood culture	AMB	Died
Song [59]	2010	Case report	South Korea	8/M	ALL, neutropenia	Fungemia, pneumonia, skin lesions	Blood culture	ITC	Died
Sparrow [60]	1992	Case report	Australia	3/M	Neuroblastoma, autoHSCT	Skin lesions, fungemia	Skin biopsy, blood, urine, endotracheal tube, faeces, throat swab culture	AMB	Died
Spielberger [61]	1995	Case report	USA	32/F	AML, AlloHSCT, neutropenia	Pneumonia, fungemia	Sputum, blood culture	AMB + ITC	Died
Stefanovic [62]	2016	Case report	Canada	44/M	Hemophagocytic lymphohistiocytosis, NHL, neutropenia	Pneumonia, fungemia	BAL, blood culture	VRC + MICA	NA
Tapia [63]	1994	Case series	Spain	45/M	MM, autoHSCT, neutropenia	Fungemia, meningism, pneumonia	Blood culture	None	Died
Tapia [63]	1994	Case series	Spain	49/M	AML, neutropenia	Pneumonia, hemiplegia	BAL culture, autopsy lung, liver, kidneys, brain (ischemic lesion) fungal invasion	AMB + ITC + Surgical resection of lung nodule	Died
Teh [64]	2019	Retrospective cohort study	Australia	68/M	CLL	Fungemia	Blood culture	CAS	Died
Tey [65]	2020	Case report	Australia	60/F	CLL, neutropenia	Fungemia, pneumonia, septic emboli brain, skin, chest	Blood culture	VRC + TRB + G-CSF	Died
Tong [66]	2007	Case report	Australia	61/M	AML, alloHSCT	Fungemia, endophthalmitis	Blood culture	CAS + VRC + TRB + intravitreal VRC	Died (no evidence of fungal infection in autopsy)
Trubiano [67]	2014	Case report	Australia	67/Μ	AML	Fungemia, endophthalmitis	Vitreous fluid, eye, temporal lobe specimen culture	CAS + VRC + TRB + intravitreal VRC + Vitrectomy/enucleation/temporal lobectomy	Survived
Valerio [68]	2021	Case report	Spain	25/Μ	Heart transplantation	Fungemia, pneumonia, skin lesions	Blood, catheter tip, tracheal aspirate, skin biopsy culture	L-AMB + VRC + TRB	Died
Whyte [69]	2005	Case report	Australia	8/F	ALL	Pneumonia, septic arthritis, osteomyelitis, discitis, epidural fluid collection	Lung biopsy, joint aspirate, laminectomy, disc debridement specimens culture	L-AMBVRC + TRB + G-CSF + Laminectomy/disc debridement/surgical joints washouts	Survived
Wilson [70]	2022	Case report	Australia	43/F	AML, neutropenia	Fungemia, pneumonia, skin lesions, septic arthritis, osteomyelitis, intracerebral lesions	Blood, synovial fluid culture	VRC + TRB + MTF + Debridement/synovectomy/arthroscopic washout	Died
Wise [71]	1993	Case report	Australia	53/M	Renal transplantation	Pneumonia, peritonitis	Peritoneal, wound swabs, pleural, ileostomy, jejunal fluid culture	AMBMIC	Died
Wood [72]	1992	Case series	Australia	52/M	AML	Fungemia, endophthalmitis	Vitreous aspirate, urine, blood, skin biopsy culture, autopsy renal abscess culture	AMB + FCS	Died
Wood [72]	1992	Case series	Australia	46/M	ALL, neutropenia	Fungemia	Blood from Hickman catheter culture	None	Died
Strickland [73]	1998	Case series	USA	51/F	Breast cancer, autoHSCT, neutropenia	Fungemia, pneumonia, pericardial effusion, pleural effusion	Blood culture, autopsy specimens (heart, lung, liver)	AMB	Died
Carreter de Granda [74]	2001	Case report	Spain	52/F	MM, BMT, neutropenia	Fungemia, endocarditis, endophthalmitis, brain mycotic aneurysm	Blood, valve specimen culture	L-AMB + ITC + Valve replacement	Died
Freeman [75]	2007	Case series	USA	24/F	Hyper IgE syndrome	Pneumonia, cerebritis	Lung tissue autopsy culture, cerebritis/ pyelonephritis with budding hyphae autopsy	AMB + POS	Died
Fernandez-Guerrero [76]	2011	Case report	Spain	29/F	ALL	Endocarditis, septic arthritis, osteomyelitis, mycotic aneurysm, endophthalmitis	Blood, vitreous fluid, embolus, valve vegetations culture	VRC + L-AMB + TRB + Embolectomy/valve replacement	Died
Kelly [77]	2016	Case report	Australia	75/F	Ovarian carcinoma	Endocarditis, cerebral emboli, fungemia	Blood culture	VRC	Died
O’ Hearn [78]	2010	Case report	USA	38/F	Heart transplantation	Endophthalmitis	Vitreous specimen, chest wall lesion culture	Intravitreal AMB/VRC + VRC + TRB + Vitrectomy	Survived
Ochi [79]	2015	Case report	Japan	66/F	AML, neutropenia	Fungemia, sinusitis, pulmonary/splenic emboli, endocarditis	Blood, sputum, CSF culture	FLCVRC + L-AMBVRC + TRB + G-CSF	Died
Ohashi [80]	2011	Case report	Japan	58/M	Monoclonal gammopathy of undermined significance	Fungemia, pneumonia	Blood, sputum culture	ITCL-AMBMICA + VRC	Died
Sayah [81]	2013	Case report	USA	70/F	Lung transplantation	Pericarditis, mycotic aneurysm, pneumonia	BAL, pericardial culture	VRC + TRB + MICA + Pericardiectomy	Died
Smita [82]	2015	Case report	India	50/M	Pacemaker implantation, diabetes	Fungemia, endocarditis	Blood, valve tissue specimen culture	L-AMBVRC + POSVRC + TRB + Valve replacement	Survived
Tascini [83]	2006	Case report	Italy	75/M	Pacemaker implantation	Endocarditis, pneumonia	Tips of the lead culture	VRC + Pacemaker removal	Survived
Uno [84]	2014	Case report	Japan	35/M	Renal transplantation	Fungemia, endocarditis, meningitis, pneumonia	Blood, sputum, CSF culture	ITRA + MICAL-AMB + VRC	Died
Wakabayashi [85]	2016	Case report	Japan	64/F	Chronic osteomyelitis	Fungemia, endocarditis, endophthalmitis, osteomyelitis	Blood culture	FLC	Died
Ahmad [86] #	2010	Case report	USA	50/M	Rheumatic disease	Fungemia, brain emboli	Blood culture	L-AMB + Valve replacement	Died
Spanevello [87]	2010	Case report	Australia	28/F	Acute undifferentiated leukemia, neutropenia	Pseudoaneurysm, cerebral hemorrhage	Blood, sinus material culture	VRC + TRB	Died
Beldarrain [88]	2000	Case report	Spain	42/F	AML, neutropenia	Fungemia, pneumonia, ischemic brain infarct	Blood culture	FLC	Died
Guadalajara [89]	2018	Case report	Spain	36/F	Multiple sclerosis, glucocorticoids	Mycotic cerebral aneurysm, ischemic stroke	Fungal structures in the arterial wall of ruptured aneurysm, thrombus, larynx, small intestine	None	Died
Tamaki [90]	2016	Case report	Japan	62/M	AML, neutropenia, alloHSCT	Meningitis, fungemia	Blood, CSF culture and PCR	MICAL-AMB + VRC	Died
Takata [91]	2020	Case report	Japan	70/F	AML	Endophthalmitis, brain aneurysm, fungemia	Blood culture, fungal structures in the arterial wall of the aneurysm	CAS + AMBVRC + Aneurysm resection	Died
Marco de Lucas [92]	2006	Case series	Spain	37/M	AML, alloHSCT, neutropenia	Orbit cellulitis, multiple brain lesions, pneumonia	Autopsy	AMB + ITC + FLC	Died
Marco de Lucas [92]	2006	Case series	Spain	66/M	AML, neutropenia	Multiple brain lesions, pneumonia	Autopsy	AMB + ITC + FLC	Died
Marco de Lucas [92]	2006	Case series	Spain	45/M	MM, alloHSCT, neutropenia	Arterial brain thrombosis, pneumonia	Blood culture, Autopsy	AMB + ITC + FLC	Died
Marco de Lucas [92]	2006	Case series	Spain	18/F	MDS, AlloHSCT, neutropenia	Pansinusitis, orbital cellulitis, multiple brain lesions, pneumonia	Autopsy	AMB + ITC + FLC	Died
Marco de Lucas [92]	2006	Case series	Spain	36/M	AML, alloHSCT, neutropenia	Multiple brain lesions, pneumonia	Autopsy	AMB + ITC + FLC	Died
Marco de Lucas [92]	2006	Case series	Spain	52/F	MM, autoHSCT, neutropenia	Endocarditis, subarachnoid hemorrhage, bilateral panuveitis, pneumonia	Blood culture	AMB + ITC + FLC	Died
Elizondo-Zertuche [93]	2017	Case series	Mexico	48/F	CML, BMT	Fungemia	BAL, urine, blood culture	ITC + CAS	Died
Elizondo-Zertuche [93]	2017	Case series	Mexico	61/M	AIDS	Fungemia	Blood culture	FLC	Died
Elizondo-Zertuche [93]	2017	Case series	Mexico	47/F	CML	Sepsis	BAL, vitreous culture	None	Died
Elizondo-Zertuche [93]	2017	Case series	Mexico	57/F	Renal transplantation	Fungemia	Blood culture	AMB	Died
Elizondo-Zertuche [93]	2017	Case series	Mexico	67/M	AML	Fungemia	Blood, peritoneal fluid culture	FLC + AMB	Died
Elizondo-Zertuche [93]	2017	Case series	Mexico	40/M	AML	Fungemia	Blood culture	AMB	Died
Idigoras [94]	2001	Case series	Spain	44/F	AML, neutropenia	Fungemia, pneumonia, conjunctival effusion, cutaneous eruption	Blood culture	AMB	Died
Idigoras [94]	2001	Case series	Spain	55/F	Breast cancer, autoHSCT, neutropenia	Fungemia	Blood culture	ITC + G-CSF	Survived
Idigoras [94]	2001	Case series	Spain	28/M	AIDS	Fungemia, pneumonia	BAL, blood, urine, feces, sputum culture	None	Died
Idigoras [94]	2001	Case series	Spain	65/M	AML, neutropenia	Fungemia, skin lesions, pneumonia	Blood, sputum culture	FLC + ITC + AMB + G-CSF	Died
Idigoras [94]	2001	Case series	Spain	56/F	AML, neutropenia	Fungemia, pneumonia	Blood culture	FLC + G-CSF	Died
Idigoras [94]	2001	Case series	Spain	28/M	AML	Fungemia, spondylodiscitis, abdominal abscess, skin lesions, cholecystitis	Blood, wound, abscess culture	FLC + ITC + AMB + G-CSF + TRB + VRC + Abscess drainage	Died
Jenks [95]	2018	Retrospective cohort study	USA	NA/NA	NHL	Fungemia	Blood culture	MICA + L-AMB	Died
Jenks [95]	2018	Retrospective cohort study	USA	NA/NA	Chronic granulomatous disease	Fungemia	Blood culture	VRC + TRB	Survived
Vagefi [96]	2005	Case report	USA	56/F	Lung transplantation	Pneumonia, endophthalmitis	Bronchial bruising, vitreous culture	VRC + TRB + intravitreal AMB/VRC	Died
Johnson [97]	2014	Retrospective cohort study	USA	54/M	Mutlivisceral transplantation	NA	Autopsy heart, pericardium, pleura, kidneys, brain	AMB + CAS + VRC	Died
Johnson [97]	2014	Retrospective cohort study	USA	51/F	Mutlivisceral transplantation	NA	Autopsy pericardium, eyes, dermis, heart, kidneys, pancreas	AMB + CAS + VRC	Died
Nasif [98]	2021	Case report	USA	48/M	Renal transplantation	Thigh, brain, shin abscesses, femoral artery mycotic aneurysm	Thigh, brain, shin abscesses culture	POS + AMB + SurgeryTRB + VRC + Surgery	Died
Tintelnot [13]	2009	Retrospective cohort study	Germany	54/F	Renal transplantation	Fungemia, pneumonia, skin lesions, sepsis	Blood culture	L-AMB + FCS + MIC	Died
Tintelnot [13]	2009	Retrospective cohort study	Germany	53/F	AML	Fungemia, pneumonia, skin lesions, endophthalmitis, sepsis	Blood culture	AMB + FCS	Died
Tintelnot [13]	2009	Retrospective cohort study	Germany	61/F	Long term corticosteroids	Fungemia, pneumonia	Blood, tracheal secretions culture	None	Died
Tintelnot [13]	2009	Retrospective cohort study	Germany	44/F	CML, BMT	Fungemia, pneumonia, sepsis	BAL, urine, catheter, blood culture	CAS	Died
Tintelnot [13]	2009	Retrospective cohort study	Germany	NA/M	BMT	Fungemia, endophthalmitis, sepsis	Blood culture	POS	Died
Tintelnot [13]	2009	Retrospective cohort study	Germany	40/M	AML	Fungemia, sepsis	Blood culture	AMB	Died
Tintelnot [13]	2009	Retrospective cohort study	Germany	64/M	AML	Fungemia, brain involvement	Blood culture	None	Died
Tintelnot [13]	2009	Retrospective cohort study	Germany	60/M	Chronic idiopathic myelofibrosis, BMT	Fungemia, sepsis	Blood, BAL culture	VRC + CAS	Died
Tintelnot [13]	2009	Retrospective cohort study	Germany	47/F	COPD, lung transplantation	Endophthalmitis, sepsis	BAL, vitreous fluid culture	POS + CASL-AMBVRC	Died
Husain [99] *	2005	Case series	USA/Spain	55/Μ	Small bowel transplantation	Peritoneum involvement	NA	AMB	Died
Husain [99] *	2005	Case series	USA/Spain	40/M	Kidney/pancreas transplantation	CNS, pulmonary involvement	NA	VRC	Survived
Husain [99] *	2005	Case series	USA/Spain	51/F	Small bowel transplantation	Aneurysm	NA	AMB + VRC + CAS	Died
Husain [99] *	2005	Case series	USA/Spain	17/M	Liver transplantation	Pulmonary involvement	NA	VRC	Died
Husain [99] *	2005	Case series	USA/Spain	44/F	Heart transplantation	Pulmonary, sinus, skin involvement	NA	AMB	Died
Husain [99] *	2005	Case series	USA/Spain	68/M	Kidney/liver transplantation	Skin involvement	NA	VRC	Survived

AML: acute myeloid leukemia, ALL: acute lymphoblastic leukemia, AMML: acute myelomonocytic leukemia, NHL: non-Hodgkin lymphoma, CML: chronic myeloid leukemia, MM: multiple myeloma, MDS: myelodysplastic syndrome, ΒΜΤ: bone marrow transplantation, AlloHSCT: allogenic hemopoietic stem cell transplantation, AutoHSCT: autologous hemopoietic stem cell transplantaton, COPD: chronic obstructive pulmonary disease, AMB: amphotericin B, L-AMB: liposomal amphotericin B, VRC: voriconazole, TRB: terbinafine, POS: posaconazole, CAS: caspofungin, MTF: miltefosine, ANF: anidulafungin, ITC: itraconazole, ALB: albaconazole, FLC: fluconazole, FCS: flucytosine, ISA: isavuconazole, MIC: miconazole, MICA: micafungin, NA: not applicable. * This study includes six solid organ recipients with *L. prolificans* infection affecting many systems, but it is not clearly stated if dissemination is present. # Information extracted from other articles [77,85].

## Supplementary Materials

The following supporting information can be downloaded at: https://www.mdpi.com/article/10.3390/pathogens12010067/s1. Table S1: Reasons for exclusion of studies from the systematic review; Table S2: Reported cases and their risk of bias according to the Joanna Briggs Institute (JBI) Critical Appraisal Checklist for Case Reports.

## References

[1] Rodriguez-Tudela J.L., Berenguer J., Guarro J., Kantarcioglu A.S., Horre R., de Hoog G.S., Cuenca-Estrella M. (2009). Epidemiology and Outcome of Scedosporium Prolificans Infection, a Review of 162 Cases. Med. Mycol..

[2] Summerbell R.C., Krajden S., Kane J. (1989). Potted Plants in Hospitals as Reservoirs of Pathogenic Fungi. Mycopathologia.

[3] Hennebert G.L. (1974). Lomentospora Prolificans, a New Hyphomycete from Greenhouse Soil. Mycotaxon.

[4] Malloch D., Salkin I.F. (1984). A New Species of Scedosporium Associated with Osteomyelitis in Humans. Mycotaxon.

[5] Chen S.C.-A., Halliday C.L., Hoenigl M., Cornely O.A., Meyer W. (2021). Scedosporium and Lomentospora Infections: Contemporary Microbiological Tools for the Diagnosis of Invasive Disease. J. Fungi.

[6] De Hoog G.S., Guarro J., Gene J., Ahmed S., Al-Hatmi A.M.S., Figueras J., Vitale R.G. (2019). Atlas of Clinical Fungi.

[7] Cortez K.J., Roilides E., Quiroz-Telles F., Meletiadis J., Antachopoulos C., Knudsen T., Buchanan W., Milanovich J., Sutton D.A., Fothergill A. (2008). Infections Caused by *Scedosporium* spp.. Clin. Microbiol. Rev..

[8] Seidel D., Meißner A., Lackner M., Piepenbrock E., Salmanton-García J., Stecher M., Mellinghoff S., Hamprecht A., Durán Graeff L., Köhler P. (2019). Prognostic Factors in 264 Adults with Invasive Scedosporium Spp. and Lomentospora Prolificans Infection Reported in the Literature and FungiScope^®^. Crit. Rev. Microbiol..

[9] Cooley L., Spelman D., Thursky K., Slavin M. (2007). Infection with Scedosporium Apiospermum and S. Prolificans, Australia. Emerg. Infect. Dis..

[10] DeSimone M.S., Crothers J.W., Solomon I.H., Laga A.C. (2021). Scedosporium and Lomentospora Infections Are Infrequent, Difficult to Diagnose by Histology, and Highly Virulent. Am. J. Clin. Pathol..

[11] Rolfe N.E., Sandin R.L., Greene J.N. (2014). Scedosporium Infections at a Cancer Center over a 10-Year Period (2001–2010). Infect. Dis. Clin. Pract..

[12] Berenguer J., Rodríguez-Tudela J.L., Richard C., Alvarez M., Sanz M.A., Gaztelurrutia L., Ayats J., Martinez-Suarez J.V. (1997). Deep Infections Caused by Scedosporium Prolificans. A Report on 16 Cases in Spain and a Review of the Literature. Scedosporium Prolificans Spanish Study Group. Medicine.

[13] Tintelnot K., Just-Nübling G., Horré R., Graf B., Sobottka I., Seibold M., Haas A., Kaben U., De Hoog G.S. (2009). A Review of German Scedosporium Prolificans Cases from 1993 to 2007. Med. Mycol..

[14] Alvarez M., Lopez Ponga B., Rayon C., Garcia Gala J., Roson Porto M.C., Gonzalez M., Martinez-Suarez J.V., Rodriguez-Tudela J.L. (1995). Nosocomial Outbreak Caused by Scedosporium Prolificans (Inflatum): Four Fatal Cases in Leukemic Patients. J. Clin. Microbiol..

[15] Cuenca-Estrella M., Alastruey-Izquierdo A., Alcazar-Fuoli L., Bernal-Martinez L., Gomez-Lopez A., Buitrago M.J., Mellado E., Rodriguez-Tudela J.L. (2008). In Vitro Activities of 35 Double Combinations of Antifungal Agents against Scedosporium Apiospermum and Scedosporium Prolificans. Antimicrob. Agents Chemother..

[16] Cuenca-Estrella M., Bassetti M., Lass-Flörl C., Rácil Z., Richardson M., Rogers T.R. (2011). Detection and Investigation of Invasive Mould Disease. J. Antimicrob. Chemother..

[17] Page M.J., McKenzie J.E., Bossuyt P.M., Boutron I., Hoffmann T.C., Mulrow C.D., Shamseer L., Tetzlaff J.M., Akl E.A., Brennan S.E. (2021). The PRISMA 2020 Statement: An Updated Guideline for Reporting Systematic Reviews. BMJ.

[18] Chapter 7: Systematic Reviews of Etiology and Risk-JBI Manual for Evidence Synthesis-JBI Global Wiki. https://jbi-global-wiki.refined.site/space/MANUAL/4687372/Chapter+7%3A+Systematic+reviews+of+etiology+and+risk.

[19] Aldoss I., Dadwal S., Zhang J., Tegtmeier B., Mei M., Arslan S., Al Malki M.M., Salhotra A., Ali H., Aribi A. (2019). Invasive Fungal Infections in Acute Myeloid Leukemia Treated with Venetoclax and Hypomethylating Agents. Blood Adv..

[20] Álvarez-Uría A., Guinea J.V., Escribano P., Gómez-Castellá J., Valerio M., Galar A., Vena A., Bouza E., Muñoz P. (2020). Invasive Scedosporium and Lomentosora Infections in the Era of Antifungal Prophylaxis: A 20-Year Experience from a Single Centre in Spain. Mycoses.

[21] Ananda-Rajah M.R., Grigg A., Slavin M.A. (2008). Breakthrough Disseminated Scedosporium Prolificans Infection in a Patient with Relapsed Leukaemia on Prolonged Voriconazole Followed by Posaconazole Prophylaxis. Mycopathologia.

[22] Balandin B., Aguilar M., Sánchez I., Monzón A., Rivera I., Salas C., Valdivia M., Alcántara S., Pérez A., Ussetti P. (2016). Scedosporium Apiospermum and S. Prolificans Mixed Disseminated Infection in a Lung Transplant Recipient: An Unusual Case of Long-Term Survival with Combined Systemic and Local Antifungal Therapy in Intensive Care Unit. Med. Mycol. Case Rep..

[23] Barbaric D., Shaw P.J. (2001). Scedosporium Infection in Immunocompromised Patients: Successful Use of Liposomal Amphotericin B and Itraconazole. Med. Pediatr. Oncol..

[24] Boan P., Pang S., Gardam D.J., Darragh H., Wright M., Coombs G.W. (2020). Investigation of a Lomentospora Prolificans Case Cluster with Whole Genome Sequencing. Med. Mycol. Case Rep..

[25] Boglione-Kerrien C., Verdier M.-C., Gautier-Veyret E., Hennart B., Belaz S., Revest M., Lemaitre F. (2019). Using Unusual Drug-Drug Interactions to Maximize Voriconazole Treatment Efficacy. Med. Mal. Infect..

[26] Bouza E., Muñoz P., Vega L., Rodríguez-Créixems M., Berenguer J., Escudero A. (1996). Clinical Resolution of Scedosporium Prolificans Fungemia Associated with Reversal of Neutropenia Following Administration of Granulocyte Colony-Stimulating Factor. Clin. Infect. Dis..

[27] Buil J.B., Pickkers P., van der Lee H.A.L., Verweij P.E. (2021). A Mould Infection in Disguise. Clin. Microbiol. Infect..

[28] Chiam N., Rose L.V.T., Waters K.D., Elder J.E. (2013). Scedosporium Prolificans Endogenous Endophthalmitis. J. AAPOS.

[29] Cobo F., Lara-Oya A., Rodríguez-Granger J., Sampedro A., Aliaga-Martínez L., Navarro-Marí J.M. (2018). Infections Caused by Scedosporium/Lomentospora Species: Clinical and Microbiological Findings in 21 Cases. Med. Mycol..

[30] Damronglerd P., Phuphuakrat A., Santanirand P., Sungkanuparph S. (2014). Disseminated Scedosporium prolificans infection in a patient with acute myeloid leukemia and prolonged febril neutropenia. J. Infect. Dis. Antimicrob. Agents.

[31] de Batlle J., Motjé M., Balanzà R., Guardia R., Ortiz R. (2000). Disseminated Infection Caused by Scedosporium Prolificans in a Patient with Acute Multilineal Leukemia. J. Clin. Microbiol..

[32] Elsayed S., Lannigan R., Chin-Yee I. (1999). Scedosporium Prolificans Fungemia. Can. J. Infect. Dis..

[33] Farag S.S., Firkin F.C., Andrew J.H., Lee C.S., Ellis D.H. (1992). Fatal Disseminated Scedosporium Inflatum Infection in a Neutropenic Immunocompromised Patient. J. Infect..

[34] Feltkamp M.C., Kersten M.J., van der Lelie J., Burggraaf J.D., de Hoog G.S., Kuijper E.J. (1997). Fatal Scedosporium Prolificans Infection in a Leukemic Patient. Eur. J. Clin. Microbiol. Infect. Dis..

[35] Gosbell I.B., Morris M.L., Gallo J.H., Weeks K.A., Neville S.A., Rogers A.H., Andrews R.H., Ellis D.H. (1999). Clinical, Pathologic and Epidemiologic Features of Infection with Scedosporium Prolificans: Four Cases and Review. Clin. Microbiol. Infect..

[36] Gow-Lee V.J., Moyers J.T., Rogstad D.K. (2021). Fatal Recurrent Disseminated Lomentospora Prolificans Infection during Autologous Hematopoietic Stem Cell Transplantation: A Case Report and Review, and Discussion on the Importance of Prolonged Neutropenia. Transpl. Infect. Dis..

[37] Grenouillet F., Botterel F., Crouzet J., Larosa F., Hicheri Y., Forel J.-M., Helias P., Ranque S., Delhaes L. (2009). Scedosporium Prolificans: An Emerging Pathogen in France?. Med. Mycol..

[38] Guerrero A., Torres P., Duran M.T., Ruiz-Díez B., Rosales M., Rodriguez-Tudela J.L. (2001). Airborne Outbreak of Nosocomial Scedosporium Prolificans Infection. Lancet.

[39] Hanmantgad M., Nog R., Seiter K. (2017). Acute Myeloid Leukemia and Fatal Scedosporium Prolificans Sepsis after Eculizumab Treatment for Paroxysmal Nocturnal Hemoglobinuria: A Case Report. Stem Cell Investig..

[40] Howden B.P., Slavin M.A., Schwarer A.P., Mijch A.M. (2003). Successful Control of Disseminated Scedosporium Prolificans Infection with a Combination of Voriconazole and Terbinafine. Eur. J. Clin. Microbiol. Infect. Dis..

[41] Jain P., Nagarajan P., Prayag P., Benton C.B., Kadia T., Groisberg R., Kontoyiannis D.P., Mulanovich V.E., Pemmaraju N. (2017). Mixed Angioinvasive Exserohilum and Scedosporium Infection in a Patient with AML. Am. J. Hematol..

[42] Kimura M., Maenishi O., Ito H., Ohkusu K. (2010). Unique Histological Characteristics of Scedosporium That Could Aid in Its Identification. Pathol. Int..

[43] Kubisiak-Rzepczyk H., Gil L., Zawirska A., Kubisiak-Michalska A., Mol A., Reich A., Komarnicki M., Adamski Z. (2013). Scedosporium Prolificans Fungaemia in a Patient with Acute Lymphoblastic Leukaemia. J. Mycol. Med..

[44] Maertens J., Lagrou K., Deweerdt H., Surmont I., Verhoef G.E., Verhaegen J., Boogaerts M.A. (2000). Disseminated Infection by Scedosporium Prolificans: An Emerging Fatality among Haematology Patients. Case Report and Review. Ann. Hematol..

[45] Marin J., Sanz M.A., Sanz G.F., Guarro J., Martínez M.L., Prieto M., Gueho E., Menezo J.L. (1991). Disseminated Scedosporium Inflatum Infection in a Patient with Acute Myeloblastic Leukemia. Eur. J. Clin. Microbiol. Infect. Dis..

[46] Westerman D.A., Speed B.R., Prince H.M. (1999). Fatal Disseminated Infection by Scedosporium Prolificans during Induction Therapy for Acute Leukemia: A Case Report and Literature Review. Pathology.

[47] McKelvie P.A., Wong E.Y., Chow L.P., Hall A.J. (2001). Scedosporium Endophthalmitis: Two Fatal Disseminated Cases of Scedosporium Infection Presenting with Endophthalmitis. Clin. Exp. Ophthalmol..

[48] Nambiar P.H., Tokarczyk M., DeSimone J.A. (2017). Answer to October 2017 Photo Quiz. J. Clin. Microbiol..

[49] Nenoff P., Gütz U., Tintelnot K., Bosse-Henck A., Mierzwa M., Hofmann J., Horn L.C., Haustein U.F. (1996). Disseminated Mycosis Due to Scedosporium Prolificans in an AIDS Patient with Burkitt Lymphoma. Mycoses.

[50] Nielsen K., Lang H., Shum A.C., Woodruff K., Cherry J.D. (1993). Disseminated Scedosporium Prolificans Infection in an Immunocompromised Adolescent. Pediatr. Infect. Dis. J..

[51] Nishimori M., Takahashi T., Suzuki E., Kodaka T., Hiramoto N., Itoh K., Tsunemine H., Yarita K., Kamei K., Takegawa H. (2014). Fatal Fungemia with Scedosporium Prolificans in a Patient with Acute Myeloid Leukemia. Med. Mycol. J..

[52] Penteado F.D., Litvinov N., Sztajnbok J., Thomaz D.Y., Dos Santos A.M., Vasconcelos D.M., Motta A.L., Rossi F., Fernandes J.F., Marques H.H.S. (2018). Lomentospora Prolificans Fungemia in Hematopoietic Stem Cell Transplant Patients: First Report in South America and Literature Review. Transpl. Infect. Dis..

[53] Pickles R.W., Pacey D.E., Muir D.B., Merrell W.H. (1996). Experience with Infection by Scedosporium Prolificans Including Apparent Cure with Fluconazole Therapy. J. Infect..

[54] Rabodonirina M., Paulus S., Thevenet F., Loire R., Gueho E., Bastien O., Mornex J.F., Celard M., Piens M.A. (1994). Disseminated Scedosporium Prolificans (S. Inflatum) Infection after Single-Lung Transplantation. Clin. Infect. Dis..

[55] Reinoso R., Carreño E., Hileeto D., Corell A., Pastor J.C., Cabrero M., Vázquez L., Calonge M. (2013). Fatal Disseminated Scedosporium Prolificans Infection Initiated by Ophthalmic Involvement in a Patient with Acute Myeloblastic Leukemia. Diagn Microbiol. Infect. Dis..

[56] Rivier A., Perny J., Debourgogne A., Thivillier C., Lévy B., Gérard A., Machouart M. (2011). Fatal Disseminated Infection Due to Scedosporium Prolificans in a Patient with Acute Myeloid Leukemia and Posaconazole Prophylaxis. Leuk. Lymphoma.

[57] Salesa R., Burgos A., Ondiviela R., Richard C., Quindos G., Ponton J. (1993). Fatal Disseminated Infection by Scedosporium Inflatum after Bone Marrow Transplantation. Scand. J. Infect. Dis..

[58] Simarro E., Marín F., Morales A., Sanz E., Pérez J., Ruiz J. (2001). Fungemia Due to Scedosporium Prolificans: A Description of Two Cases with Fatal Outcome. Clin. Microbiol. Infect..

[59] Song M.J., Lee J.H., Lee N.Y. (2011). Fatal Scedosporium Prolificans Infection in a Paediatric Patient with Acute Lymphoblastic Leukaemia. Mycoses.

[60] Sparrow S.A., Hallam L.A., Wild B.E., Baker D.L. (1992). Scedosporium Inflatum: First Case Report of Disseminated Infection and Review of the Literature. Pediatr. Hematol. Oncol..

[61] Spielberger R.T., Tegtmeier B.R., O’Donnell M.R., Ito J.I. (1995). Fatal Scedosporium Prolificans (S. Inflatum) Fungemia Following Allogeneic Bone Marrow Transplantation: Report of a Case in the United States. Clin. Infect. Dis..

[62] Stefanovic A., Wright A., Tang V., Hoang L. (2016). Positive Blood Cultures in a Patient Recovering from Febrile Neutropenia. JMM Case Rep..

[63] Tapia M., Richard C., Baro J., Salesa R., Figols J., Zurbano F., Zubizarreta A. (1994). Scedosporium Inflatum Infection in Immunocompromised Haematological Patients. Br. J. Haematol..

[64] Teh B.W., Chui W., Handunnetti S., Tam C., Worth L.J., Thursky K.A., Slavin M.A. (2019). High Rates of Proven Invasive Fungal Disease with the Use of Ibrutinib Monotherapy for Relapsed or Refractory Chronic Lymphocytic Leukemia. Leuk. Lymphoma.

[65] Tey A., Mohan B., Cheah R., Dendle C., Gregory G. (2020). Disseminated Lomentospora Prolificans Infection in a Patient on Idelalisib-Rituximab Therapy for Relapsed Chronic Lymphocytic Leukaemia. Ann. Hematol..

[66] Tong S.Y.C., Peleg A.Y., Yoong J., Handke R., Szer J., Slavin M. (2007). Breakthrough Scedosporium Prolificans Infection While Receiving Voriconazole Prophylaxis in an Allogeneic Stem Cell Transplant Recipient. Transpl. Infect. Dis..

[67] Trubiano J.A., Paratz E., Wolf M., Teh B.W., Todaro M., Thursky K.A., Slavin M.A. (2014). Disseminated Scedosporium Prolificans Infection in an “Extensive Metaboliser”: Navigating the Minefield of Drug Interactions and Pharmacogenomics. Mycoses.

[68] Valerio M., Vásquez V., Álvarez-Uria A., Zatarain-Nicolás E., Pavone P., Martínez-Jiménez M.D.C., Barrio-Gutiérrez J.M., Cuerpo G., Guinea-Ortega J., Vena A. (2021). Disseminated Lomentosporiosis in a Heart Transplant Recipient: Case Report and Review of the Literature. Transpl. Infect. Dis..

[69] Whyte M., Irving H., O’Regan P., Nissen M., Siebert D., Labrom R. (2005). Disseminated Scedosporium Prolificans Infection and Survival of a Child with Acute Lymphoblastic Leukemia. Pediatr. Infect. Dis. J..

[70] Wilson P.A., MacKenzie S. (2022). Disseminated Lomentospora Prolificans Infection in a Patient With Acute Myeloid Leukemia Salvage Therapy With Miltefosine. Infect. Dis. Clin. Pract..

[71] Wise K.A., Speed B.R., Ellis D.H., Andrew J.H. (1993). Two Fatal Infections in Immunocompromised Patients Caused by Scedosporium Inflatum. Pathology.

[72] Wood G.M., McCormack J.G., Muir D.B., Ellis D.H., Ridley M.F., Pritchard R., Harrison M. (1992). Clinical Features of Human Infection with Scedosporium Inflatum. Clin. Infect. Dis..

[73] Strickland L.B., Sandin R.L., Greene J.N., Ahmad N. (1998). A Breast Cancer Patient with Disseminated Scedosporium Prolificans Infection. Infect. Med..

[74] Carreter de Granda M.E., Richard C., Conde E., Iriondo A., Marco de Lucas F., Salesa R., Zubizarreta A. (2001). Endocarditis Caused by Scedosporium Prolificans after Autologous Peripheral Blood Stem Cell Transplantation. Eur. J. Clin. Microbiol. Infect. Dis..

[75] Freeman A.F., Kleiner D.E., Nadiminti H., Davis J., Quezado M., Anderson V., Puck J.M., Holland S.M. (2007). Causes of Death in Hyper-IgE Syndrome. J. Allergy Clin. Immunol..

[76] Fernandez Guerrero M.L., Askari E., Prieto E., Gadea I., Román A. (2011). Emerging Infectious Endocarditis Due to Scedosporium Prolificans: A Model of Therapeutic Complexity. Eur. J. Clin. Microbiol. Infect. Dis..

[77] Kelly M., Stevens R., Konecny P. (2016). Lomentospora Prolificans Endocarditis--Case Report and Literature Review. BMC Infect. Dis..

[78] OʼHearn T.M., Geiseler P.J., Bhatti R.A., Eliott D. (2010). Control of Disseminated Scedosporium Prolificans Infection and Endophthalmitis. Retin. Cases Brief Rep..

[79] Ochi Y., Hiramoto N., Takegawa H., Yonetani N., Doi A., Ichikawa C., Imai Y., Ishikawa T. (2015). Infective Endocarditis Caused by Scedosporium Prolificans Infection in a Patient with Acute Myeloid Leukemia Undergoing Induction Chemotherapy. Int. J. Hematol..

[80] Ohashi R., Kato M., Katsura Y., Takekawa H., Hoshika Y., Sugawara T., Yoshimi K., Togo S., Nagaoka T., Seyama K. (2011). Breakthrough Lung Scedosporium Prolificans Infection with Multiple Cavity Lesions in a Patient Receiving Voriconazole for Probable Invasive Aspergillosis Associated with Monoclonal Gammopathy of Undetermined Significance (MGUS). Med. Mycol. J..

[81] Sayah D.M., Schwartz B.S., Kukreja J., Singer J.P., Golden J.A., Leard L.E. (2013). Scedosporium Prolificans Pericarditis and Mycotic Aortic Aneurysm in a Lung Transplant Recipient Receiving Voriconazole Prophylaxis. Transpl. Infect. Dis..

[82] Smita S., Sunil S., Amarjeet K., Anil B., Yatin M. (2015). Surviving a Recurrent Scedosporium Prolificans Endocarditis: Mention If Consent Was Taken. Indian J. Med. Microbiol..

[83] Tascini C., Bongiorni M.G., Leonildi A., Giannola G., Soldati E., Arena G., Doria R., Germenia C., Menichetti F. (2006). Pacemaker Endocarditis with Pulmonary Cavitary Lesion Due to Scedosporium Prolificans. J. Chemother..

[84] Uno K., Kasahara K., Kutsuna S., Katanami Y., Yamamoto Y., Maeda K., Konishi M., Ogawa T., Yoneda T., Yoshida K. (2014). Infective Endocarditis and Meningitis Due to Scedosporium Prolificans in a Renal Transplant Recipient. J. Infect. Chemother..

[85] Wakabayashi Y., Okugawa S., Tatsuno K., Ikeda M., Misawa Y., Koyano S., Tsuji E., Yanagimoto S., Hatakeyama S., Moriya K. (2016). Scedosporium Prolificans Endocarditis: Case Report and Literature Review. Intern. Med..

[86] Ahmad S., Zia S., Sarwari A.R. (2010). Scedosporium Prolificans Endocarditis: Case Report and Review of Literature. W. Va. Med. J..

[87] Spanevello M., Morris K.L., Kennedy G.A. (2010). Pseudoaneurysm Formation by Scedosporium Prolificans Infection in Acute Leukaemia. Intern. Med. J..

[88] Gomez Beldarrain M., Garca-Monco J.C., Ojanguren J., Zabalza I., De Miguel E. (2000). Scedosporum Prolificans Infection: An Unusual Cause of Cerebral Infarct [3]. Am. J. Med..

[89] Guadalajara M.C.V., Hernández González A., Carrasco García de León S., Rojo M.G., Del Real Francia M.Á. (2018). Mycotic Cerebral Aneurysms Secondary to Scedosporium Prolificans Infection in a Patient with Multiple Sclerosis. J. Clin. Neurol..

[90] Tamaki M., Nozaki K., Onishi M., Yamamoto K., Ujiie H., Sugahara H. (2016). Fungal Meningitis Caused by Lomentospora Prolificans after Allogeneic Hematopoietic Stem Cell Transplantation. Transpl. Infect. Dis..

[91] Takata S., Tamase A., Hayashi Y., Anzawa K., Shioya A., Iinuma Y., Iizuka H. (2020). Ruptured Fungal Aneurysm of the Peripheral Middle Cerebral Artery Caused by Lomentospora Infection: A Case Report and Literature Review. Interdiscip. Neurosurg. Adv. Tech. Case Manag..

[92] Marco de Lucas E., Sádaba P., Lastra García-Barón P., Ruiz Delgado M.L., Cuevas J., Salesa R., Bermúdez A., González Mandly A., Gutiérrez A., Fernández F. (2006). Cerebral Scedosporiosis: An Emerging Fungal Infection in Severe Neutropenic Patients: CT Features and CT Pathologic Correlation. Eur. Radiol..

[93] Elizondo-Zertuche M., Montoya A.M., Robledo-Leal E., Garza-Veloz I., Sánchez-Núñez A.L., Ballesteros-Elizondo R., González G.M. (2017). Comparative Pathogenicity of Lomentospora Prolificans (Scedosporium Prolificans) Isolates from Mexican Patients. Mycopathologia.

[94] Idigoras P., Pérez-Trallero E., Piñeiro L., Larruskain J., López-Lopategui M.C., Rodríguez N., González J.M. (2001). Disseminated Infection and Colonization by Scedosporium Prolificans: A Review of 18 Cases, 1990-1999. Clin. Infect. Dis..

[95] Jenks J.D., Reed S.L., Seidel D., Koehler P., Cornely O.A., Mehta S.R., Hoenigl M. (2018). Rare Mould Infections Caused by Mucorales, Lomentospora Prolificans and Fusarium, in San Diego, CA: The Role of Antifungal Combination Therapy. Int. J. Antimicrob. Agents.

[96] Vagefi M.R., Kim E.T., Alvarado R.G., Duncan J.L., Howes E.L., Crawford J.B. (2005). Bilateral Endogenous Scedosporium Prolificans Endophthalmitis after Lung Transplantation. Am. J. Ophthalmol..

[97] Johnson L.S., Shields R.K., Clancy C.J. (2014). Epidemiology, Clinical Manifestations, and Outcomes of Scedosporium Infections among Solid Organ Transplant Recipients. Transpl. Infect. Dis..

[98] Nasif A., Siebenaller D., DeRiso A., Shah H., Alharthi S., Nazzal M. (2021). Disseminated Lomentospora Prolificans Infection Presenting with Arterial Exsanguination. J. Vasc. Surg. Cases Innov. Tech..

[99] Husain S., Muñoz P., Forrest G., Alexander B.D., Somani J., Brennan K., Wagener M.M., Singh N. (2005). Infections Due to Scedosporium Apiospermum and Scedosporium Prolificans in Transplant Recipients: Clinical Characteristics and Impact of Antifungal Agent Therapy on Outcome. Clin. Infect. Dis..

[100] Konsoula A., Tsioutis C., Markaki I., Papadakis M., Agouridis A.P., Spernovasilis N. (2022). Lomentospora Prolificans: An Emerging Opportunistic Fungal Pathogen. Microorganisms.

[101] Daniele L., Le M., Parr A.F., Brown L.M. (2017). Scedosporium Prolificans Septic Arthritis and Osteomyelitis of the Hip Joints in an Immunocompetent Patient: A Case Report and Literature Review. Case Rep. Orthop..

[102] Vazirani J., Westall G.P., Snell G.I., Morrissey C.O. (2021). Scedosporium Apiospermum and Lomentospora Prolificans in Lung Transplant Patients-A Single Center Experience over 24 Years. Transpl. Infect. Dis..

[103] Tamm M., Malouf M., Glanville A. (2001). Pulmonary Scedosporium Infection Following Lung Transplantation. Transpl. Infect. Dis..

[104] Pianalto K.M., Alspaugh J.A. (2016). New Horizons in Antifungal Therapy. J. Fungi.

[105] Jenks J.D., Seidel D., Cornely O.A., Chen S., van Hal S., Kauffman C., Miceli M.H., Heinemann M., Christner M., Jover Sáenz A. (2020). Clinical Characteristics and Outcomes of Invasive Lomentospora Prolificans Infections: Analysis of Patients in the FungiScope^®^ Registry. Mycoses.

[106] Bronnimann D., Garcia-Hermoso D., Dromer F., Lanternier F., French Mycoses Study Group (2021). Characterization of the isolates at the NRCMA Scedosporiosis/Lomentosporiosis Observational Study (SOS): Clinical Significance of Scedosporium Species Identification. Med. Mycol..

[107] Seidel D., Hassler A., Salmanton-García J., Koehler P., Mellinghoff S.C., Carlesse F., Cheng M.P., Falces-Romero I., Herbrecht R., Jover Sáenz A. (2020). Invasive Scedosporium Spp. and Lomentospora Prolificans Infections in Pediatric Patients: Analysis of 55 Cases from FungiScope® and the Literature. Int. J. Infect. Dis..

[108] de Mello T.P., Aor A.C., de Oliveira S.S.C., Branquinha M.H., Santos A.L.S.D. (2016). Conidial Germination in Scedosporium Apiospermum, S. Aurantiacum, S. Minutisporum and Lomentospora Prolificans: Influence of Growth Conditions and Antifungal Susceptibility Profiles. Mem. Inst. Oswaldo Cruz.

[109] Mello T.P., Aor A.C., Gonçalves D.S., Seabra S.H., Branquinha M.H., Santos A.L.S. (2016). Assessment of Biofilm Formation by Scedosporium Apiospermum, S. Aurantiacum, S. Minutisporum and Lomentospora Prolificans. Biofouling.

[110] Kauffman C.A. (2006). Fungal Infections. Proc. Am. Thorac. Soc..

[111] Rollin-Pinheiro R., da Silva Xisto M.I.D., Rochetti V.P., Barreto-Bergter E. (2020). Scedosporium Cell Wall: From Carbohydrate-Containing Structures to Host-Pathogen Interactions. Mycopathologia.

[112] Pellon A., Ramirez-Garcia A., Guruceaga X., Zabala A., Buldain I., Antoran A., Anguita J., Rementeria A., Matute C., Hernando F.L. (2018). Microglial Immune Response Is Impaired against the Neurotropic Fungus Lomentospora Prolificans. Cell Microbiol..

[113] Donnelly J.P., Chen S.C., Kauffman C.A., Steinbach W.J., Baddley J.W., Verweij P.E., Clancy C.J., Wingard J.R., Lockhart S.R., Groll A.H. (2020). Revision and Update of the Consensus Definitions of Invasive Fungal Disease From the European Organization for Research and Treatment of Cancer and the Mycoses Study Group Education and Research Consortium. Clin. Infect. Dis..

[114] Buldain I., Martin-Souto L., Antoran A., Areitio M., Aparicio-Fernandez L., Rementeria A., Hernando F.L., Ramirez-Garcia A. (2021). The Host Immune Response to Scedosporium/Lomentospora. J. Fungi.

[115] Lau A., Chen S., Sorrell T., Carter D., Malik R., Martin P., Halliday C. (2007). Development and Clinical Application of a Panfungal PCR Assay to Detect and Identify Fungal DNA in Tissue Specimens. J. Clin. Microbiol..

[116] Buitrago M.J., Bernal-Martinez L., Castelli M.V., Rodriguez-Tudela J.L., Cuenca-Estrella M. (2014). Performance of Panfungal- and Specific-PCR-Based Procedures for Etiological Diagnosis of Invasive Fungal Diseases on Tissue Biopsy Specimens with Proven Infection: A 7-Year Retrospective Analysis from a Reference Laboratory. J. Clin. Microbiol..

[117] Ruiz-Díez B., Martín-Díez F., Rodríguez-Tudela J.L., Alvárez M., Martínez-Suárez J.V. (1997). Use of Random Amplification of Polymorphic DNA (RAPD) and PCR-Fingerprinting for Genotyping a Scedosporium Prolificans (Inflatum) Outbreak in Four Leukemic Patients. Curr. Microbiol..

[118] Odabasi Z., Paetznick V.L., Rodriguez J.R., Chen E., McGinnis M.R., Ostrosky-Zeichner L. (2006). Differences in Beta-Glucan Levels in Culture Supernatants of a Variety of Fungi. Med. Mycol..

[119] Wilkendorf L.S., Bowles E., Buil J.B., van der Lee H.A.L., Posteraro B., Sanguinetti M., Verweij P.E. (2020). Update on Matrix-Assisted Laser Desorption Ionization-Time of Flight Mass Spectrometry Identification of Filamentous Fungi. J. Clin. Microbiol..

[120] Zvezdanova M.E., Escribano P., Ruiz A., Martínez-Jiménez M.C., Peláez T., Collazos A., Guinea J., Bouza E., Rodríguez-Sánchez B. (2019). Increased Species-Assignment of Filamentous Fungi Using MALDI-TOF MS Coupled with a Simplified Sample Processing and an in-House Library. Med. Mycol..

[121] Hoenigl M., Salmanton-García J., Walsh T.J., Nucci M., Neoh C.F., Jenks J.D., Lackner M., Sprute R., Al-Hatmi A.M.S., Bassetti M. (2021). Global Guideline for the Diagnosis and Management of Rare Mould Infections: An Initiative of the European Confederation of Medical Mycology in Cooperation with the International Society for Human and Animal Mycology and the American Society for Microbiology. Lancet Infect. Dis..

[122] Jenks J.D., Seidel D., Cornely O.A., Chen S., van Hal S., Kauffman C., Miceli M.H., Heinemann M., Christner M., Jover Sáenz A. (2020). Voriconazole plus Terbinafine Combination Antifungal Therapy for Invasive Lomentospora Prolificans Infections: Analysis of 41 Patients from the FungiScope^®^ Registry 2008–2019. Clin. Microbiol. Infect..

[123] Hoenigl M., Sprute R., Egger M., Arastehfar A., Cornely O.A., Krause R., Lass-Flörl C., Prattes J., Spec A., Thompson G.R. (2021). The Antifungal Pipeline: Fosmanogepix, Ibrexafungerp, Olorofim, Opelconazole, and Rezafungin. Drugs.

[124] Wiederhold N.P. (2020). Review of the Novel Investigational Antifungal Olorofim. J. Fungi.

[125] Chen S., Rai N.J., Cunneen S., Cornelissen K., Rex J.H., Heath C.H., Harvey E. A Case of Lomentospora Prolificans Treated with the Novel Antifungal Olorofim. Proceedings of the 30th European Congress of Clinical Microbiology and Infectious Diseases.

[126] Tio S.H., Thursky K., Ng G., Rex J.H., Carney D., Slavin M. Olorofim for a Case of Severe Disseminated Lomentospora Prolificans Infections. Proceedings of the 30th European Congress of Clinical Microbiology and Infectious Diseases.

