# Supplementary material for: Lomentospora prolificans Disseminated Infections: A Systematic Review of Reported Cases
Source: Pathogens. 2022 Dec 31;12(1):67. doi: 10.3390/pathogens12010067 (PMC9861501; doi:10.3390/pathogens12010067)
Supplement: Supplementary file 1 [file pathogens-12-00067-s001.zip › pathogens-2096400-supplementary.pdf]

Lomentospora prolificans Disseminated Infections: A Systematic Review of Reported Cases

Supplementary Material

Table S1. Reasons for exclusion of studies from the systematic review	
**Study**	**Reasons of Exclusion**
Praetorius [1]	Different language
Erles [2]	Animal study
Haynes [3]	Animal study
Salkin [4]	Animal study
Taylor [5]	Animal study
Fernández-Mosteirín [6]	Different language
Guarro [7]	Different language
Idígoras [8]	Different language
Idígoras [9]	Different language
Nishio [10]	Different language
Romero-Gomez [11]	Different language
Nenoff [12]	Different language
Lopez [13]	Different language

Table S2. Reported cases and their risk of bias according to the Joanna Briggs Institute (JBI) Critical Appraisal Checklist for Case Reports [14]											
**Author**	**Study type**	**Q1**	**Q2**	**Q3**	**Q4**	**Q5**	**Q6**	**Q7**	**Q8**	**Raw score and %**	**Risk**
Aldoss (2019) [15]	Retrospective cohort study	1	0	0	0	0	0	0	1	2/8 = 25%	high
Ahmad (2010) [16]	Case report	NA	NA	NA	NA	NA	NA	NA	NA	0/8 = 0%	high
Alvarez (1995) [17]	Case series	1	1	1	1	1	1	1	1	8/8 = 100%	low
Álvarez-Uría (2020) [18]	Case series	1	0	0	1	1	1	1	1	6/8 = 75%	low
Ananda-Rajah (2008) [19]	Case report	1	1	1	1	1	1	1	1	8/8 = 100%	low
Balandin (2016) [20]	Case report	1	1	1	1	1	1	1	1	8/8 = 100%	low
Barbaric (2001) [21]	Case series	1	1	1	1	1	1	1	1	8/8 = 100%	low
Beldarrain (2000) [22]	Case report	1	1	1	1	1	1	1	1	8/8 = 100%	low
Berenguer (1997) [23]	Case series	1	0	0	1	0	0	1	1	4/8 = 50%	moderate
Boan (2020) [24]	Case series	1	1	1	1	1	1	1	1	8/8 = 100%	low
Boglione-Kerriena (2019) [25]	Case report	0	1	1	1	1	1	1	1	7/8 = 87.5%	high
Bouza (1996) [26]	Case report	1	1	1	1	1	1	1	1	8/8 = 100%	low
Buil (2020) [27]	Case report	0	1	0	1	0	0	1	0	3/8 = 37.5%	high
Carreter de Granda (2001) [28]	Case report	1	1	1	1	1	1	1	1	8/8 = 100%	low
Chiam (2013) [29]	Case report	1	1	1	1	1	1	1	1	8/8 = 100%	low
Cobo (2017) [30]	Retrospective cohort study	1	0	0	0	0	0	1	1	3/8 = 37.5%	high
Cooley (2007) [31]	Case series	0	0	1	1	1	1	1	1	6/8 = 75%	low
Damronglerd (2014) [32]	Case report	1	1	1	1	1	1	1	1	8/8 = 100%	low
de Battle (2000) [33]	Case report	1	1	1	1	1	1	1	1	8/8 = 100%	low
DeSimone (2021) [34]	Retrospective cohort study	1	0	0	1	0	0	1	1	4/8 = 50%	moderate
Elizondo-Zertuche (2017) [35]	Case series	1	0	0	0	0	0	1	1	3/8 = 37.5%	high
Elsayed (1999) [36]	Case report	1	1	1	1	1	1	1	1	8/8 = 100%	low
Farag (1992) [37]	Case report	1	1	1	1	1	1	1	1	8/8 = 100%	low
Feltkamp (1997) [38]	Case report	1	1	1	1	1	1	1	1	8/8 = 100%	low
Guerrero (2011) [39]	Case report	1	1	1	1	1	1	1	1	8/8 = 100%	low
Freeman (2007) [40]	Case series	1	0	1	1	1	1	1	1	7/8 = 87.5%	low
Gosbell (1999) [41]	Case series	1	1	1	1	1	1	1	1	8/8 = 100%	low
Gow-Lee (2021) [42]	Case report	1	1	1	1	1	1	1	1	8/8 = 100%	low
Grenouillet (2009) [43]	Case series	1	1	1	1	1	1	1	1	8/8 = 100%	low
Guadalajara (2018) [44]	Case report	1	1	1	1	1	1	1	1	8/8 = 100%	low
Guerrero (2001) [45]	Case series	1	0	1	1	1	1	1	1	7/8 = 87.5%	low
Hanmantgad (2017) [46]	Case report	1	1	1	1	0	1	1	1	7/8 = 87.5%	low

Howden (2003) [47]	Case report	1	1	1	1	1	1	1	1	8/8 = 100%	low
Husain (2005) [48]	Case series	1	0	0	0	1	0	1	1	4/8 = 50%	moderate
Idigoras (2001) [49]	Case series	1	1	1	1	1	1	1	1	8/8 = 100%	low
Jain (2017) [50]	Case report	1	1	1	1	1	1	1	1	8/8 = 100%	low
Jenks (2018) [51]	Retrospective cohort study	1	0	0	0	0	0	1	1	3/8 = 37.5%	high
Johnson (2014) [52]	Retrospective cohort study	1	0	0	0	0	0	1	1	3/8 = 37.5%	high
Kelly (2016) [53]	Case report	1	1	1	1	1	1	1	1	8/8 = 100%	low
Kimura (2010) [54]	Case report	1	1	1	1	1	1	1	1	8/8 = 100%	low
Kubisiak-Rzepczyk (2013) [55]	Case report	1	1	1	1	1	1	1	1	8/8 = 100%	low
Maertens (2000) [56]	Case report	1	1	1	1	1	1	1	1	8/8 = 100%	low
Marco de Lucas (2006) [57]	Case series	1	0	0	0	0	0	1	1	3/8 = 37.5%	high
Marin (1991) [58]	Case report	1	1	1	1	1	1	1	1	8/8 = 100%	low
McKelvie (2001) [59]	Case report	1	1	1	1	1	1	1	1	8/8 = 100%	low
Nambiar (2017) [60]	Case report	1	1	1	1	1	1	1	1	8/8 = 100%	low
Nasif (2021) [61]	Case report	1	1	1	1	1	1	1	1	8/8 = 100%	low
Nenoff (1996) [62]	Case report	1	1	1	1	1	1	1	1	8/8 = 100%	low
Nielsen (1993) [63]	Case report	1	1	1	1	1	1	1	1	8/8 = 100%	low
Nishimori (2014) [64]	Case report	1	1	1	1	1	1	1	1	8/8 = 100%	low
O' Hearn (2010) [65]	Case report	1	1	1	1	1	1	1	1	8/8 = 100%	low
Ochi (2015) [66]	Case report	1	1	1	1	1	1	1	1	8/8 = 100%	low
Ohashi (2011) [67]	Case report	1	1	1	1	1	1	1	1	8/8 = 100%	low
Penteado (2018) [68]	Case report	1	1	1	1	1	1	1	1	8/8 = 100%	low
Pickles (1996) [69]	Case series	1	1	1	1	1	1	1	1	8/8 = 100%	low
Rabodonirina (1994) [70]	Case report	1	1	1	1	1	1	1	1	8/8 = 100%	low
Reinoso (2013) [71]	Case report	1	1	1	1	1	1	1	1	8/8 = 100%	low
Rivier (2011) [72]	Case report	1	1	1	1	1	1	1	1	8/8 = 100%	low
Rolfe (2014) [73]	Case series	1	0	0	1	0	0	1	1	4/8 = 50%	moderate
Salesa (1993) [74]	Case report	1	1	1	1	1	1	1	1	8/8 = 100%	low
Sayah (2013) [75]	Case report	1	1	1	1	1	1	1	1	8/8 = 100%	low
Simarro (2001) [76]	Case report	1	1	1	1	1	1	1	1	8/8 = 100%	low
Smita (2015) [77]	Case report	1	1	1	1	1	1	1	1	8/8 = 100%	low
Song (2010) [78]	Case report	1	1	1	1	1	1	1	1	8/8 = 100%	low
Spanevello (2010) [79]	Case report	1	1	1	1	1	1	1	1	8/8 = 100%	low
Sparrow (1992) [80]	Case report	1	1	1	1	1	1	1	1	8/8 = 100%	low

Spielberger (1995) [81]	Case report	1	1	1	1	1	1	1	1	8/8 = 100%	low
Stefanovic (2016) [82]	Case report	1	1	1	1	1	1	0	1	7/8 = 87.5%	low
Strickland (1998) [83]	Case series	1	1	1	1	1	1	1	1	8/8 = 100%	low
Takata (2020) [84]	Case report	1	1	1	1	1	1	1	1	8/8 = 100%	low
Tamaki (2016) [85]	Case report	1	1	1	1	1	1	1	1	8/8 = 100%	low
Tapia (1994) [86]	Case series	1	1	1	1	1	1	1	1	8/8 = 100%	low
Tascini (2006) [87]	Case report	1	1	1	1	1	1	1	1	8/8 = 100%	low
Teh (2019) [88]	Retrospective cohort study	1	0	0	1	0	0	1	1	4/8 = 50%	moderate
Tey (2020) [89]	Case report	1	1	1	1	1	1	1	1	8/8 = 100%	low
Tintelnot (2009) [90]	Retrospective cohort study	1	0	0	1	0	0	1	1	4/8 = 50%	moderate
Tong (2007) [91]	Case report	1	1	1	1	1	1	1	1	8/8 = 100%	low
Trubiano (2014) [92]	Case report	1	1	1	1	1	1	1	1	8/8 = 100%	low
Uno (2014) [93]	Case report	1	1	1	1	1	1	1	1	8/8 = 100%	low
Vagefi (2005) [94]	Case report	1	1	1	1	1	1	1	1	8/8 = 100%	low
Valerio (2021) [95]	Case report	1	1	1	1	1	1	1	1	8/8 = 100%	low
Wakabayashi (2016) [96]	Case report	1	1	1	1	1	1	1	1	8/8 = 100%	low
Westerman (1999) [97]	Case report	1	1	1	1	1	1	1	1	8/8 = 100%	low
Whyte (2005) [98]	Case report	1	1	1	1	1	1	1	1	8/8 = 100%	low
Wilson (2022) [99]	Case report	1	1	1	1	1	1	1	1	8/8 = 100%	low
Wise (1993) [100]	Case report	1	1	1	1	1	1	1	1	8/8 = 100%	low
Wood (1992) [101]	Case series	1	1	1	1	1	1	1	1	8/8 = 100%	low

JBI Criteria to be scored: Q1. Were patient’s demographic characteristics clearly described? Q2. Was the patient’s history clearly described and presented as a timeline? Q3. Was the current clinical condition of the patient on presentation clearly described? Q4. Were diagnostic tests or assessment methods and the results clearly described? Q5. Was the intervention(s) or treatment procedure(s) clearly described? Q6. Was the post-intervention clinical condition clearly described? Q7. Were adverse events (harms) or unanticipated events identified and described? Q8. Does the case report provide takeaway lessons? 1 = Yes, 0 = No, and U = unclear

Abbreviationss: 1 = Yes, 0 = No, and U = unclear; NA = not applicable

Criteria used to rank the risk of bias

i. ≤ 49% = high risk of bias
ii. 50% and 69% = moderate risk of bias
iii. ≥ 70% = low risk of bias

References

1. González Praetorius, A.; del Palacio, A.; Pérez Pomata, M.T.; Díaz, M.; Pérez Simón, M.; Alén, M.J.; Gimeno, C.; Bisquert, J. [Fungemia caused by Scedosporium prolificans (inflatum) in a patient with AIDS]. *Enferm Infecc Microbiol Clin* **1998**, *16*, 382–383.
2. Erles, K.; Mugford, A.; Barfield, D.; Leeb, T.; Kook, P.H. Systemic Scedosporium Prolificans Infection in an 11-Month-Old Border Collie with Cobalamin Deficiency Secondary to Selective Cobalamin Malabsorption (Canine Imerslund-Gräsbeck Syndrome). *J Small Anim Pract* **2018**, *59*, 253–256, doi:10.1111/jsap.12678.
3. Haynes, S.M.; Hodge, P.J.; Tyrrell, D.; Abraham, L.A. Disseminated Scedosporium Prolificans Infection in a German Shepherd Dog. *Aust Vet J* **2012**, *90*, 34–38, doi:10.1111/j.1751-0813.2011.00870.x.
4. Salkin, I.F.; Cooper, C.R.; Bartges, J.W.; Kemna, M.E.; Rinaldi, M.G. Scedosporium Inflatum Osteomyelitis in a Dog. *J Clin Microbiol* **1992**, *30*, 2797–2800, doi:10.1128/jcm.30.11.2797-2800.1992.
5. Taylor, A.; Talbot, J.; Bennett, P.; Martin, P.; Makara, M.; Barrs, V.R. Disseminated Scedosporium Prolificans Infection in a Labrador Retriever with Immune Mediated Haemolytic Anaemia. *Med Mycol Case Rep* **2014**, *6*, 66–69, doi:10.1016/j.mmcr.2014.10.001.
6. Fernández-Mosteirín, N.; Salvador-Osuna, C.; Mayayo, P.; García-Zueco, J.C. [Scedosporium prolificans: disseminated infection in immunocompromised patient]. *Med Clin (Barc)* **2003**, *120*, 317–318, doi:10.1016/s0025-7753(03)73687-2.
7. Guarro, J.; Gaztelurrutia, L.; Marín, J.; Bárcena, J. [Scedosporium inflatum, a new pathogenic fungus. Report of 2 cases with a fatal outcome]. *Enferm Infecc Microbiol Clin* **1991**, *9*, 557–560.
8. Idigoras, P.; Lopez Lopategui, C.; Garcia-Arenzana, J.M.; Diaz De Tuesta, J.L.; Marin Gonzalez, J. Disseminated Infection by Scedosporium Inflatum [1]. *Enfermedades Infecciosas y Microbiologia Clinica* **1993**, *11*, 285.
9. Idigoras, P.; García-Arenzana, J.M.; Sáenz, J.R.; Piñeiro, L.; Marín, J. [Isolation of Scedosporium prolificans from the air in the room of a patient with leukemia and disseminated infection with this fungus]. *Enferm Infecc Microbiol Clin* **2000**, *18*, 426–427.
10. Nishio, H.; Utsumi, T.; Nakamura, Y.; Suzuki, T.; Kamei, K.; Saitoh, T. [Fungemia caused by Scedosporium prolificans in myelodysplastic syndrome]. *Kansenshogaku Zasshi* **2012**, *86*, 22–26, doi:10.11150/kansenshogakuzasshi.86.22.
11. Romero Gómez, M.P.; García Rodríguez, J. [Invasive fungal infection in a patient with Burkitt lymphoma]. *Rev Iberoam Micol* **2010**, *27*, 214–215, doi:10.1016/j.riam.2010.07.001.
12. Nenoff, P.; Horn, L.C.; Schwenke, H.; Mierzwa, M.; Rieske, K.; Haustein, U.F. [Invasive mold infections in the university clinics of Leipzig in the period from 1992-1994]. *Mycoses* **1996**, *39 Suppl 1*, 107–112, doi:10.1111/j.1439-0507.1996.tb00515.x.
13. López, L.; Gaztelurrutia, L.; Cuenca-Estrella, M.; Monzón, A.; Barrón, J.; Hernández, J.L.; Pérez, R. [Infection and colonization by Scedosporium prolificans]. *Enferm Infecc Microbiol Clin* **2001**, *19*, 308–313, doi:10.1016/s0213-005x(01)72651-1.
14. Chapter 7: Systematic Reviews of Etiology and Risk - JBI Manual for Evidence Synthesis - JBI Global Wiki Available online: https://jbi-global-

wiki.refined.site/space/MANUAL/4687372/Chapter+7%3A+Systematic+reviews+of+etiology+and+risk (accessed on 15 November 2022).
15. Aldoss, I.; Dadwal, S.; Zhang, J.; Tegtmeier, B.; Mei, M.; Arslan, S.; Al Malki, M.M.; Salhotra, A.; Ali, H.; Aribi, A.; et al. Invasive Fungal Infections in Acute Myeloid Leukemia Treated with Venetoclax and Hypomethylating Agents. *Blood Advances* **2019**, *3*, 4043–4049, doi:10.1182/bloodadvances.2019000930.
16. Ahmad, S.; Zia, S.; Sarwari, A.R. Scedosporium Prolificans Endocarditis: Case Report and Review of Literature. *W V Med J* **2010**, *106*, 24–26.
17. Alvarez, M.; Lopez Ponga, B.; Rayon, C.; Garcia Gala, J.; Roson Porto, M.C.; Gonzalez, M.; Martinez-Suarez, J.V.; Rodriguez-Tudela, J.L. Nosocomial Outbreak Caused by Scedosporium Prolificans (Inflatum): Four Fatal Cases in Leukemic Patients. *J Clin Microbiol* **1995**, *33*, 3290–3295, doi:10.1128/jcm.33.12.3290-3295.1995.
18. Álvarez-Uría, A.; Guinea, J.V.; Escribano, P.; Gómez-Castellá, J.; Valerio, M.; Galar, A.; Vena, A.; Bouza, E.; Muñoz, P. Invasive Scedosporium and Lomentosora Infections in the Era of Antifungal Prophylaxis: A 20-Year Experience from a Single Centre in Spain. *Mycoses* **2020**, doi:10.1111/myc.13154.
19. Ananda-Rajah, M.R.; Grigg, A.; Slavin, M.A. Breakthrough Disseminated Scedosporium Prolificans Infection in a Patient with Relapsed Leukaemia on Prolonged Voriconazole Followed by Posaconazole Prophylaxis. *Mycopathologia* **2008**, *166*, 83–86, doi:10.1007/s11046-008-9131-2.
20. Balandin, B.; Aguilar, M.; Sánchez, I.; Monzón, A.; Rivera, I.; Salas, C.; Valdivia, M.; Alcántara, S.; Pérez, A.; Ussetti, P. Scedosporium Apiospermum and S. Prolificans Mixed Disseminated Infection in a Lung Transplant Recipient: An Unusual Case of Long-Term Survival with Combined Systemic and Local Antifungal Therapy in Intensive Care Unit. *Med Mycol Case Rep* **2016**, *11*, 53–56, doi:10.1016/j.mmcr.2016.04.006.
21. Barbaric, D.; Shaw, P.J. Scedosporium Infection in Immunocompromised Patients: Successful Use of Liposomal Amphotericin B and Itraconazole. *Med Pediatr Oncol* **2001**, *37*, 122–125, doi:10.1002/mpo.1180.
22. Gomez Beldarrain, M.; Garca-Monco, J.C.; Ojanguren, J.; Zabalza, I.; De Miguel, E. Scedosporum Prolificans Infection: An Unusual Cause of Cerebral Infarct [3]. *American Journal of Medicine* **2000**, *108*, 679–680, doi:10.1016/S0002-9343(99)00400-3.
23. Berenguer, J.; Rodríguez-Tudela, J.L.; Richard, C.; Alvarez, M.; Sanz, M.A.; Gaztelurrutia, L.; Ayats, J.; Martinez-Suarez, J.V. Deep Infections Caused by Scedosporium Prolificans. A Report on 16 Cases in Spain and a Review of the Literature. Scedosporium Prolificans Spanish Study Group. *Medicine (Baltimore)* **1997**, *76*, 256–265, doi:10.1097/00005792-199707000-00004.
24. Boan, P.; Pang, S.; Gardam, D.J.; Darragh, H.; Wright, M.; Coombs, G.W. Investigation of a Lomentospora Prolificans Case Cluster with Whole Genome Sequencing. *Med Mycol Case Rep* **2020**, *29*, 1–4, doi:10.1016/j.mmcr.2020.05.003.
25. Boglione-Kerrien, C.; Verdier, M.-C.; Gautier-Veyret, E.; Hennart, B.; Belaz, S.; Revest, M.; Lemaitre, F. Using Unusual Drug-Drug Interactions to Maximize Voriconazole Treatment Efficacy. *Med Mal Infect* **2019**, *49*, 555–557, doi:10.1016/j.medmal.2019.05.004.
26. Bouza, E.; Muñoz, P.; Vega, L.; Rodríguez-Créixems, M.; Berenguer, J.; Escudero, A. Clinical Resolution of Scedosporium Prolificans Fungemia Associated with Reversal of Neutropenia Following Administration of Granulocyte Colony-Stimulating Factor. *Clin Infect Dis* **1996**, *23*, 192–193, doi:10.1093/clinids/23.1.192.

27. Buil, J.B.; Pickkers, P.; van der Lee, H.A.L.; Verweij, P.E. A Mould Infection in Disguise. *Clin Microbiol Infect* **2021**, *27*, 854–855, doi:10.1016/j.cmi.2020.11.020.
28. Carreter de Granda, M.E.; Richard, C.; Conde, E.; Iriondo, A.; Marco de Lucas, F.; Salesa, R.; Zubizarreta, A. Endocarditis Caused by Scedosporium Prolificans after Autologous Peripheral Blood Stem Cell Transplantation. *Eur J Clin Microbiol Infect Dis* **2001**, *20*, 215–217, doi:10.1007/pl00011256.
29. Chiam, N.; Rose, L.V.T.; Waters, K.D.; Elder, J.E. Scedosporium Prolificans Endogenous Endophthalmitis. *J AAPOS* **2013**, *17*, 627–629, doi:10.1016/j.jaapos.2013.07.010.
30. Cobo, F.; Lara-Oya, A.; Rodríguez-Granger, J.; Sampedro, A.; Aliaga-Martínez, L.; Navarro-Marí, J.M. Infections Caused by Scedosporium/Lomentospora Species: Clinical and Microbiological Findings in 21 Cases. *Med Mycol* **2018**, *56*, 917–925, doi:10.1093/mmy/myx147.
31. Cooley, L.; Spelman, D.; Thursky, K.; Slavin, M. Infection with Scedosporium Apiospermum and S. Prolificans, Australia. *Emerg Infect Dis* **2007**, *13*, 1170–1177, doi:10.3201/eid1308.060576.
32. Damronglerd, P.; Phuphuakrat, A.; Santanirand, P.; Sungkanuparph, S. Disseminated Scedosporium prolificans infection in a patient with acute myeloid leukemia and prolonged febril neutropenia. *J Infect Dis Antimicrob Agents* **2014**, *31*, 101–105.
33. de Batlle, J.; Motjé, M.; Balanzà, R.; Guardia, R.; Ortiz, R. Disseminated Infection Caused by Scedosporium Prolificans in a Patient with Acute Multilineal Leukemia. *J Clin Microbiol* **2000**, *38*, 1694–1695, doi:10.1128/JCM.38.4.1694-1695.2000.
34. DeSimone, M.S.; Crothers, J.W.; Solomon, I.H.; Laga, A.C. Scedosporium and Lomentospora Infections Are Infrequent, Difficult to Diagnose by Histology, and Highly Virulent. *Am J Clin Pathol* **2021**, *156*, 1044–1057, doi:10.1093/ajcp/aqab070.
35. Elizondo-Zertuche, M.; Montoya, A.M.; Robledo-Leal, E.; Garza-Veloz, I.; Sánchez-Núñez, A.L.; Ballesteros-Elizondo, R.; González, G.M. Comparative Pathogenicity of Lomentospora Prolificans (Scedosporium Prolificans) Isolates from Mexican Patients. *Mycopathologia* **2017**, *182*, 681–689, doi:10.1007/s11046-017-0137-5.
36. Elsayed, S.; Lannigan, R.; Chin-Yee, I. Scedosporium Prolificans Fungemia. *Can J Infect Dis* **1999**, *10*, 75–76, doi:10.1155/1999/635193.
37. Farag, S.S.; Firkin, F.C.; Andrew, J.H.; Lee, C.S.; Ellis, D.H. Fatal Disseminated Scedosporium Inflatum Infection in a Neutropenic Immunocompromised Patient. *J Infect* **1992**, *25*, 201–204, doi:10.1016/0163-4453(92)94104-6.
38. Feltkamp, M.C.; Kersten, M.J.; van der Lelie, J.; Burggraaf, J.D.; de Hoog, G.S.; Kuijper, E.J. Fatal Scedosporium Prolificans Infection in a Leukemic Patient. *Eur J Clin Microbiol Infect Dis* **1997**, *16*, 460–464, doi:10.1007/BF02471912.
39. Fernandez Guerrero, M.L.; Askari, E.; Prieto, E.; Gadea, I.; Román, A. Emerging Infectious Endocarditis Due to Scedosporium Prolificans: A Model of Therapeutic Complexity. *Eur J Clin Microbiol Infect Dis* **2011**, *30*, 1321–1324, doi:10.1007/s10096-011-1212-3.
40. Freeman, A.F.; Kleiner, D.E.; Nadiminti, H.; Davis, J.; Quezado, M.; Anderson, V.; Puck, J.M.; Holland, S.M. Causes of Death in Hyper-IgE Syndrome. *J Allergy Clin Immunol* **2007**, *119*, 1234–1240, doi:10.1016/j.jaci.2006.12.666.
41. Gosbell, I.B.; Morris, M.L.; Gallo, J.H.; Weeks, K.A.; Neville, S.A.; Rogers, A.H.; Andrews, R.H.; Ellis, D.H. Clinical, Pathologic and Epidemiologic Features of Infection with Scedosporium Prolificans: Four Cases and Review. *Clinical Microbiology and Infection* **1999**, *5*, 672–686, doi:10.1111/j.1469-0691.1999.tb00513.x.

42. Gow-Lee, V.J.; Moyers, J.T.; Rogstad, D.K. Fatal Recurrent Disseminated Lomentospora Prolificans Infection during Autologous Hematopoietic Stem Cell Transplantation: A Case Report and Review, and Discussion on the Importance of Prolonged Neutropenia. *Transpl Infect Dis* **2021**, *23*, e13701, doi:10.1111/tid.13701.
43. Grenouillet, F.; Botterel, F.; Crouzet, J.; Larosa, F.; Hicheri, Y.; Forel, J.-M.; Helias, P.; Ranque, S.; Delhaes, L. Scedosporium Prolificans: An Emerging Pathogen in France? *Med Mycol* **2009**, *47*, 343–350, doi:10.1080/13693780802454761.
44. Guadalajara, M.C.V.; Hernández González, A.; Carrasco García de León, S.; Rojo, M.G.; Del Real Francia, M.Á. Mycotic Cerebral Aneurysms Secondary to Scedosporium Prolificans Infection in a Patient with Multiple Sclerosis. *J Clin Neurol* **2018**, *14*, 601–603, doi:10.3988/jcn.2018.14.4.601.
45. Guerrero, A.; Torres, P.; Duran, M.T.; Ruiz-Díez, B.; Rosales, M.; Rodriguez-Tudela, J.L. Airborne Outbreak of Nosocomial Scedosporium Prolificans Infection. *Lancet* **2001**, *357*, 1267–1268, doi:10.1016/S0140-6736(00)04423-8.
46. Hanmantgad, M.; Nog, R.; Seiter, K. Acute Myeloid Leukemia and Fatal Scedosporium Prolificans Sepsis after Eculizumab Treatment for Paroxysmal Nocturnal Hemoglobinuria: A Case Report. *Stem Cell Investig* **2017**, *4*, 100, doi:10.21037/sci.2017.12.04.
47. Howden, B.P.; Slavin, M.A.; Schwarer, A.P.; Mijch, A.M. Successful Control of Disseminated Scedosporium Prolificans Infection with a Combination of Voriconazole and Terbinafine. *Eur J Clin Microbiol Infect Dis* **2003**, *22*, 111–113, doi:10.1007/s10096-002-0877-z.
48. Husain, S.; Muñoz, P.; Forrest, G.; Alexander, B.D.; Somani, J.; Brennan, K.; Wagener, M.M.; Singh, N. Infections Due to Scedosporium Apiospermum and Scedosporium Prolificans in Transplant Recipients: Clinical Characteristics and Impact of Antifungal Agent Therapy on Outcome. *Clin Infect Dis* **2005**, *40*, 89–99, doi:10.1086/426445.
49. Idigoras, P.; Pérez-Trallero, E.; Piñeiro, L.; Larruskain, J.; López-Lopategui, M.C.; Rodríguez, N.; González, J.M. Disseminated Infection and Colonization by Scedosporium Prolificans: A Review of 18 Cases, 1990-1999. *Clin Infect Dis* **2001**, *32*, E158-165, doi:10.1086/320521.
50. Jain, P.; Nagarajan, P.; Prayag, P.; Benton, C.B.; Kadia, T.; Groisberg, R.; Kontoyiannis, D.P.; Mulanovich, V.E.; Pemmaraju, N. Mixed Angioinvasive Exserohilum and Scedosporium Infection in a Patient with AML. *Am J Hematol* **2017**, *92*, 119–120, doi:10.1002/ajh.24455.
51. Jenks, J.D.; Reed, S.L.; Seidel, D.; Koehler, P.; Cornely, O.A.; Mehta, S.R.; Hoenigl, M. Rare Mould Infections Caused by Mucorales, Lomentospora Prolificans and Fusarium, in San Diego, CA: The Role of Antifungal Combination Therapy. *Int J Antimicrob Agents* **2018**, *52*, 706–712, doi:10.1016/j.ijantimicag.2018.08.005.
52. Johnson, L.S.; Shields, R.K.; Clancy, C.J. Epidemiology, Clinical Manifestations, and Outcomes of Scedosporium Infections among Solid Organ Transplant Recipients. *Transpl Infect Dis* **2014**, *16*, 578–587, doi:10.1111/tid.12244.
53. Kelly, M.; Stevens, R.; Konecny, P. Lomentospora Prolificans Endocarditis--Case Report and Literature Review. *BMC Infect Dis* **2016**, *16*, 36, doi:10.1186/s12879-016-1372-y.
54. Kimura, M.; Maenishi, O.; Ito, H.; Ohkusu, K. Unique Histological Characteristics of Scedosporium That Could Aid in Its Identification. *Pathol Int* **2010**, *60*, 131–136, doi:10.1111/j.1440-1827.2009.02491.x.

55. Kubisiak-Rzepczyk, H.; Gil, L.; Zawirska, A.; Kubisiak-Michalska, A.; Mol, A.; Reich, A.; Komarnicki, M.; Adamski, Z. Scedosporium Prolificans Fungaemia in a Patient with Acute Lymphoblastic Leukaemia. *J Mycol Med* **2013**, *23*, 261–264, doi:10.1016/j.mycmed.2013.08.003.
56. Maertens, J.; Lagrou, K.; Deweerdt, H.; Surmont, I.; Verhoef, G.E.; Verhaegen, J.; Boogaerts, M.A. Disseminated Infection by Scedosporium Prolificans: An Emerging Fatality among Haematology Patients. Case Report and Review. *Ann Hematol* **2000**, *79*, 340–344, doi:10.1007/s002779900137.
57. Marco de Lucas, E.; Sádaba, P.; Lastra García-Barón, P.; Ruiz Delgado, M.L.; Cuevas, J.; Salesa, R.; Bermúdez, A.; González Mandly, A.; Gutiérrez, A.; Fernández, F.; et al. Cerebral Scedosporiosis: An Emerging Fungal Infection in Severe Neutropenic Patients: CT Features and CT Pathologic Correlation. *Eur Radiol* **2006**, *16*, 496–502, doi:10.1007/s00330-005-2869-8.
58. Marin, J.; Sanz, M.A.; Sanz, G.F.; Guarro, J.; Martínez, M.L.; Prieto, M.; Gueho, E.; Menezo, J.L. Disseminated Scedosporium Inflatum Infection in a Patient with Acute Myeloblastic Leukemia. *Eur J Clin Microbiol Infect Dis* **1991**, *10*, 759–761, doi:10.1007/BF01972505.
59. McKelvie, P.A.; Wong, E.Y.; Chow, L.P.; Hall, A.J. Scedosporium Endophthalmitis: Two Fatal Disseminated Cases of Scedosporium Infection Presenting with Endophthalmitis. *Clin Exp Ophthalmol* **2001**, *29*, 330–334, doi:10.1046/j.1442-9071.2001.00444.x.
60. Nambiar, P.H.; Tokarczyk, M.; DeSimone, J.A. Answer to October 2017 Photo Quiz. *J Clin Microbiol* **2017**, *55*, 3149–3150, doi:10.1128/JCM.02488-15.
61. Nasif, A.; Siebenaller, D.; DeRiso, A.; Shah, H.; Alharthi, S.; Nazzal, M. Disseminated Lomentospora Prolificans Infection Presenting with Arterial Exsanguination. *J Vasc Surg Cases Innov Tech* **2021**, *7*, 785–789, doi:10.1016/j.jvscit.2021.08.009.
62. Nenoff, P.; Gütz, U.; Tintelnot, K.; Bosse-Henck, A.; Mierzwa, M.; Hofmann, J.; Horn, L.C.; Haustein, U.F. Disseminated Mycosis Due to Scedosporium Prolificans in an AIDS Patient with Burkitt Lymphoma. *Mycoses* **1996**, *39*, 461–465, doi:10.1111/j.1439-0507.1996.tb00098.x.
63. Nielsen, K.; Lang, H.; Shum, A.C.; Woodruff, K.; Cherry, J.D. Disseminated Scedosporium Prolificans Infection in an Immunocompromised Adolescent. *Pediatr Infect Dis J* **1993**, *12*, 882–884, doi:10.1097/00006454-199310000-00018.
64. Nishimori, M.; Takahashi, T.; Suzuki, E.; Kodaka, T.; Hiramoto, N.; Itoh, K.; Tsunemine, H.; Yarita, K.; Kamei, K.; Takegawa, H.; et al. Fatal Fungemia with Scedosporium Prolificans in a Patient with Acute Myeloid Leukemia. *Med Mycol J* **2014**, *55*, E63-70, doi:10.3314/mmj.55.E63.
65. O'Hearn, T.M.; Geiseler, P.J.; Bhatti, R.A.; Eliott, D. Control of Disseminated Scedosporium Prolificans Infection and Endophthalmitis. *Retin Cases Brief Rep* **2010**, *4*, 18–19, doi:10.1097/ICB.0b013e318196b27e.
66. Ochi, Y.; Hiramoto, N.; Takegawa, H.; Yonetani, N.; Doi, A.; Ichikawa, C.; Imai, Y.; Ishikawa, T. Infective Endocarditis Caused by Scedosporium Prolificans Infection in a Patient with Acute Myeloid Leukemia Undergoing Induction Chemotherapy. *Int J Hematol* **2015**, *101*, 620–625, doi:10.1007/s12185-015-1752-x.
67. Ohashi, R.; Kato, M.; Katsura, Y.; Takekawa, H.; Hoshika, Y.; Sugawara, T.; Yoshimi, K.; Togo, S.; Nagaoka, T.; Seyama, K.; et al. Breakthrough Lung Scedosporium Prolificans Infection with Multiple Cavity Lesions in a Patient Receiving Voriconazole for Probable

Invasive Aspergillosis Associated with Monoclonal Gammopathy of Undetermined Significance (MGUS). *Med Mycol J* **2011**, *52*, 33–38, doi:10.3314/jjmm.52.33.
68. Penteado, F.D.; Litvinov, N.; Sztajnbok, J.; Thomaz, D.Y.; Dos Santos, A.M.; Vasconcelos, D.M.; Motta, A.L.; Rossi, F.; Fernandes, J.F.; Marques, H.H.S.; et al. Lomentospora Prolificans Fungemia in Hematopoietic Stem Cell Transplant Patients: First Report in South America and Literature Review. *Transpl Infect Dis* **2018**, *20*, e12908, doi:10.1111/tid.12908.
69. Pickles, R.W.; Pacey, D.E.; Muir, D.B.; Merrell, W.H. Experience with Infection by Scedosporium Prolificans Including Apparent Cure with Fluconazole Therapy. *J Infect* **1996**, *33*, 193–197, doi:10.1016/s0163-4453(96)92249-5.
70. Rabodonirina, M.; Paulus, S.; Thevenet, F.; Loire, R.; Gueho, E.; Bastien, O.; Mornex, J.F.; Celard, M.; Piens, M.A. Disseminated Scedosporium Prolificans (S. Inflatum) Infection after Single-Lung Transplantation. *Clin Infect Dis* **1994**, *19*, 138–142, doi:10.1093/clinids/19.1.138.
71. Reinoso, R.; Carreño, E.; Hileeto, D.; Corell, A.; Pastor, J.C.; Cabrero, M.; Vázquez, L.; Calonge, M. Fatal Disseminated Scedosporium Prolificans Infection Initiated by Ophthalmic Involvement in a Patient with Acute Myeloblastic Leukemia. *Diagn Microbiol Infect Dis* **2013**, *76*, 375–378, doi:10.1016/j.diagmicrobio.2013.03.006.
72. Rivier, A.; Perny, J.; Debourgogne, A.; Thivillier, C.; Lévy, B.; Gérard, A.; Machouart, M. Fatal Disseminated Infection Due to Scedosporium Prolificans in a Patient with Acute Myeloid Leukemia and Posaconazole Prophylaxis. *Leuk Lymphoma* **2011**, *52*, 1607–1610, doi:10.3109/10428194.2011.573034.
73. Rolfe, N.E.; Sandin, R.L.; Greene, J.N. Scedosporium Infections at a Cancer Center over a 10-Year Period (2001-2010). *Infectious Diseases in Clinical Practice* **2014**, *22*, 71–74, doi:10.1097/IPC.0b013e318297d5bf.
74. Salesa, R.; Burgos, A.; Ondiviela, R.; Richard, C.; Quindos, G.; Ponton, J. Fatal Disseminated Infection by Scedosporium Inflatum after Bone Marrow Transplantation. *Scand J Infect Dis* **1993**, *25*, 389–393, doi:10.3109/00365549309008516.
75. Sayah, D.M.; Schwartz, B.S.; Kukreja, J.; Singer, J.P.; Golden, J.A.; Leard, L.E. Scedosporium Prolificans Pericarditis and Mycotic Aortic Aneurysm in a Lung Transplant Recipient Receiving Voriconazole Prophylaxis. *Transpl Infect Dis* **2013**, *15*, E70-74, doi:10.1111/tid.12056.
76. Simarro, E.; Marín, F.; Morales, A.; Sanz, E.; Pérez, J.; Ruiz, J. Fungemia Due to Scedosporium Prolificans: A Description of Two Cases with Fatal Outcome. *Clin Microbiol Infect* **2001**, *7*, 645–647, doi:10.1046/j.1198-743x.2001.00317.x.
77. Smita, S.; Sunil, S.; Amarjeet, K.; Anil, B.; Yatin, M. Surviving a Recurrent Scedosporium Prolificans Endocarditis: Mention If Consent Was Taken. *Indian J Med Microbiol* **2015**, *33*, 588–590, doi:10.4103/0255-0857.167322.
78. Song, M.J.; Lee, J.H.; Lee, N.Y. Fatal Scedosporium Prolificans Infection in a Paediatric Patient with Acute Lymphoblastic Leukaemia. *Mycoses* **2011**, *54*, 81–83, doi:10.1111/j.1439-0507.2009.01765.x.
79. Spanevello, M.; Morris, K.L.; Kennedy, G.A. Pseudoaneurysm Formation by Scedosporium Prolificans Infection in Acute Leukaemia. *Intern Med J* **2010**, *40*, 793, doi:10.1111/j.1445-5994.2010.02355.x.

80. Sparrow, S.A.; Hallam, L.A.; Wild, B.E.; Baker, D.L. Scedosporium Inflatum: First Case Report of Disseminated Infection and Review of the Literature. *Pediatr Hematol Oncol* **1992**, *9*, 293–295, doi:10.3109/08880019209016600.
81. Spielberger, R.T.; Tegtmeier, B.R.; O'Donnell, M.R.; Ito, J.I. Fatal Scedosporium Prolificans (S. Inflatum) Fungemia Following Allogeneic Bone Marrow Transplantation: Report of a Case in the United States. *Clin Infect Dis* **1995**, *21*, 1067, doi:10.1093/clinids/21.4.1067.
82. Stefanovic, A.; Wright, A.; Tang, V.; Hoang, L. Positive Blood Cultures in a Patient Recovering from Febrile Neutropenia. *JMM Case Rep* **2016**, *3*, e005038, doi:10.1099/jmmcr.0.005038.
83. Strickland, L.B.; Sandin, R.L.; Greene, J.N.; Ahmad, N. A Breast Cancer Patient with Disseminated Scedosporium Prolificans Infection. *Infections in Medicine* **1998**, *15*.
84. Takata, S.; Tamase, A.; Hayashi, Y.; Anzawa, K.; Shioya, A.; Iinuma, Y.; Iizuka, H. Ruptured Fungal Aneurysm of the Peripheral Middle Cerebral Artery Caused by Lomentospora Infection: A Case Report and Literature Review. *Interdisciplinary Neurosurgery: Advanced Techniques and Case Management* **2020**, *21*, doi:10.1016/j.inat.2020.100743.
85. Tamaki, M.; Nozaki, K.; Onishi, M.; Yamamoto, K.; Ujiie, H.; Sugahara, H. Fungal Meningitis Caused by Lomentospora Prolificans after Allogeneic Hematopoietic Stem Cell Transplantation. *Transpl Infect Dis* **2016**, *18*, 601–605, doi:10.1111/tid.12563.
86. Tapia, M.; Richard, C.; Baro, J.; Salesa, R.; Figols, J.; Zurbano, F.; Zubizarreta, A. Scedosporium Inflatum Infection in Immunocompromised Haematological Patients. *Br J Haematol* **1994**, *87*, 212–214, doi:10.1111/j.1365-2141.1994.tb04897.x.
87. Tascini, C.; Bongiorni, M.G.; Leonildi, A.; Giannola, G.; Soldati, E.; Arena, G.; Doria, R.; Germenia, C.; Menichetti, F. Pacemaker Endocarditis with Pulmonary Cavitary Lesion Due to Scedosporium Prolificans. *J Chemother* **2006**, *18*, 667–669, doi:10.1179/joc.2006.18.6.667.
88. Teh, B.W.; Chui, W.; Handunnetti, S.; Tam, C.; Worth, L.J.; Thursky, K.A.; Slavin, M.A. High Rates of Proven Invasive Fungal Disease with the Use of Ibrutinib Monotherapy for Relapsed or Refractory Chronic Lymphocytic Leukemia. *Leukemia and Lymphoma* **2019**, *60*, 1572–1575, doi:10.1080/10428194.2018.1543884.
89. Tey, A.; Mohan, B.; Cheah, R.; Dendle, C.; Gregory, G. Disseminated Lomentospora Prolificans Infection in a Patient on Idelalisib-Rituximab Therapy for Relapsed Chronic Lymphocytic Leukaemia. *Ann Hematol* **2020**, *99*, 2455–2456, doi:10.1007/s00277-020-04087-2.
90. Tintelnot, K.; Just-Nübling, G.; Horré, R.; Graf, B.; Sobottka, I.; Seibold, M.; Haas, A.; Kaben, U.; De Hoog, G.S. A Review of German Scedosporium Prolificans Cases from 1993 to 2007. *Med Mycol* **2009**, *47*, 351–358, doi:10.1080/13693780802627440.
91. Tong, S.Y.C.; Peleg, A.Y.; Yoong, J.; Handke, R.; Szer, J.; Slavin, M. Breakthrough Scedosporium Prolificans Infection While Receiving Voriconazole Prophylaxis in an Allogeneic Stem Cell Transplant Recipient. *Transpl Infect Dis* **2007**, *9*, 241–243, doi:10.1111/j.1399-3062.2007.00203.x.
92. Trubiano, J.A.; Paratz, E.; Wolf, M.; Teh, B.W.; Todaro, M.; Thursky, K.A.; Slavin, M.A. Disseminated Scedosporium Prolificans Infection in an "Extensive Metaboliser": Navigating the Minefield of Drug Interactions and Pharmacogenomics. *Mycoses* **2014**, *57*, 572–576, doi:10.1111/myc.12199.

93. Uno, K.; Kasahara, K.; Kutsuna, S.; Katanami, Y.; Yamamoto, Y.; Maeda, K.; Konishi, M.; Ogawa, T.; Yoneda, T.; Yoshida, K.; et al. Infective Endocarditis and Meningitis Due to Scedosporium Prolificans in a Renal Transplant Recipient. *J Infect Chemother* **2014**, *20*, 131–133, doi:10.1016/j.jiac.2013.09.006.
94. Vagefi, M.R.; Kim, E.T.; Alvarado, R.G.; Duncan, J.L.; Howes, E.L.; Crawford, J.B. Bilateral Endogenous Scedosporium Prolificans Endophthalmitis after Lung Transplantation. *Am J Ophthalmol* **2005**, *139*, 370–373, doi:10.1016/j.ajo.2004.08.005.
95. Valerio, M.; Vásquez, V.; Álvarez-Uria, A.; Zatarain-Nicolás, E.; Pavone, P.; Martínez-Jiménez, M.D.C.; Barrio-Gutiérrez, J.M.; Cuerpo, G.; Guinea-Ortega, J.; Vena, A.; et al. Disseminated Lomentosporiosis in a Heart Transplant Recipient: Case Report and Review of the Literature. *Transpl Infect Dis* **2021**, *23*, e13574, doi:10.1111/tid.13574.
96. Wakabayashi, Y.; Okugawa, S.; Tatsuno, K.; Ikeda, M.; Misawa, Y.; Koyano, S.; Tsuji, E.; Yanagimoto, S.; Hatakeyama, S.; Moriya, K.; et al. Scedosporium Prolificans Endocarditis: Case Report and Literature Review. *Intern Med* **2016**, *55*, 79–82, doi:10.2169/internalmedicine.55.5592.
97. Westerman, D.A.; Speed, B.R.; Prince, H.M. Fatal Disseminated Infection by Scedosporium Prolificans during Induction Therapy for Acute Leukemia: A Case Report and Literature Review. *Pathology* **1999**, *31*, 393–394, doi:10.1080/003130299104783.
98. Whyte, M.; Irving, H.; O'Regan, P.; Nissen, M.; Siebert, D.; Labrom, R. Disseminated Scedosporium Prolificans Infection and Survival of a Child with Acute Lymphoblastic Leukemia. *Pediatr Infect Dis J* **2005**, *24*, 375–377, doi:10.1097/01.inf.0000157213.94392.30.
99. Wilson, P.A.; MacKenzie, S. Disseminated Lomentospora Prolificans Infection in a Patient With Acute Myeloid Leukemia Salvage Therapy With Miltefosine. *Infectious Diseases in Clinical Practice* **2022**, *30*, doi:10.1097/IPC.0000000000001103.
100. Wise, K.A.; Speed, B.R.; Ellis, D.H.; Andrew, J.H. Two Fatal Infections in Immunocompromised Patients Caused by Scedosporium Inflatum. *Pathology* **1993**, *25*, 187–189, doi:10.3109/00313029309084797.
101. Wood, G.M.; McCormack, J.G.; Muir, D.B.; Ellis, D.H.; Ridley, M.F.; Pritchard, R.; Harrison, M. Clinical Features of Human Infection with Scedosporium Inflatum. *Clin Infect Dis* **1992**, *14*, 1027–1033, doi:10.1093/clinids/14.5.1027.
